# EAF2 mediates germinal centre B-cell apoptosis to suppress excessive immune responses and prevent autoimmunity

**DOI:** 10.1038/ncomms10836

**Published:** 2016-03-03

**Authors:** Yingqian Li, Yoshimasa Takahashi, Shin-ichiro Fujii, Yang Zhou, Rongjian Hong, Akari Suzuki, Takeshi Tsubata, Koji Hase, Ji-Yang Wang

**Affiliations:** 1 Department of Immunology, School of Basic Medical Sciences, Fudan University, Shanghai 200032, China; 2 Division of Mucosal Barriology, International Research and Development Center for Mucosal Vaccines, The Institute of Medical Science, The University of Tokyo, Tokyo 142-0054, Japan; 3 Department of Biochemistry, Faculty of Pharmacy, Keio University, Tokyo 105-8512, Japan; 4 Department of Immunology, National Institute of Infectious Diseases, Tokyo 162-8640, Japan; 5 Laboratory for Immunotherapy, Center for Integrative Medical Sciences, RIKEN, Yokohama 230-0045, Japan; 6 Laboratory for Autoimmune Diseases, Center for Integrative Medical Sciences, RIKEN, Yokohama 230-0045, Japan; 7 Department of Immunology, Medical Research Institute, Tokyo Medical and Dental University, Tokyo 113-8510, Japan

## Abstract

Regulated apoptosis of germinal centre (GC) B cells is critical for normal humoral immune responses. ELL-associated factor 2 (EAF2) regulates transcription elongation and has been shown to be an androgen-responsive potential tumour suppressor in prostate by inducing apoptosis. Here we show that EAF2 is selectively upregulated in GC B cells among various immune cell types and promotes apoptosis of GC B cells both *in vitro* and *in vivo.* EAF2 deficiency results in enlarged GCs and elevated antibody production during a T-dependent immune response. After immunization with type II collagen, mice lacking EAF2 produce high levels of collagen-specific autoantibodies and rapidly develop severe arthritis. Moreover, the mutant mice spontaneously produce anti-dsDNA, rheumatoid factor and anti-nuclear antibodies as they age. These results demonstrate that EAF2-mediated apoptosis in GC B cells limits excessive humoral immune responses and is important for maintaining self-tolerance.

## Abstract (web summary)

EAF2 has been previously known as a transcriptional elongation factor and a proapoptotic gene lost in prostate cancer. Here the authors show that EAF2 is required for apoptosis of germinal centre B cells, and that EAF2-deficient mice develop excessive antibody responses and autoimmunity.

## Graphical abstract

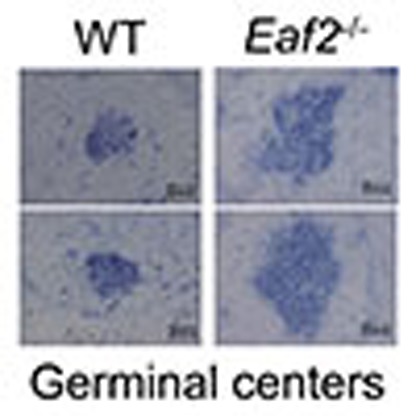

Germinal centre (GC) B cells represent a unique cell population that is induced during an adaptive immune response. These rapidly dividing cells undergo Ig gene somatic hypermutation (SHM) and class switch recombination, and those with high affinity for the foreign antigen (Ag) are selected to differentiate into plasma cells or memory B cells. Studies thus far indicate that regulated apoptosis of GC B cells is important for appropriate GC formation and optimal humoral immune responses^1^. In addition, apoptosis is thought to be involved in the elimination of self-reactive GC B cells^2^,^3^,^4^,^5^, which can be generated by SHM (refs 5, 6, 7, 8). Two principal signalling pathways initiate apoptosis in GC B cells^9^,^10^. The intrinsic pathway is regulated by Bcl-2 family members such as *Bcl-2* (refs 11, 12), *Bcl-xL* (ref. 13) and *Mcl-1* (ref. 14). On the other hand, the extrinsic pathway is activated when death receptors such as FAS (CD95) on the B-cell surface are engaged by cognate ligands of the tumour necrosis factor family^15^,^16^,^17^.

To identify GC B-cell-specific apoptosis inducer that contributes to the normal humoral immune response and the elimination of self-reactive GC B cells, we searched for apoptosis-related genes highly expressed in GC B cells. We compared gene expression profiles of a variety of different immune cell subpopulation and found the ELL (eleven-nineteen lysine-rich leukaemia)-associated factor 2 (*Eaf2*) to be highly expressed in GC B cells but not in many other immune cell types including various T cell and dendritic cell subsets. The EAF family members EAF1 and EAF2 were originally identified as novel proteins interacting with ELL, a fusion partner of the multi-lineage leukaemia gene in the t(11;19)(q23;p13.1) recurrent chromosomal translocation associated with acute myeloid leukaemia^18^,^19^. *U19*, an androgen-responsive gene in the prostate^20^, was independently shown to encode EAF2 (ref. 21). By binding to ELL, EAF1 and EAF2/U19 efficiently stimulate ELL elongation activity^22^. *In vitro* and *in vivo* functional assays have revealed that EAF2/U19 induces growth arrest and apoptosis of prostate cancer cells^21^,^23^. *Eaf2/U19*-knockout mice on a C57BL/6J background developed prostatic intraepithelial neoplasia^24^. Collectively, these observations suggested a critical role for EAF2 in the induction of apoptosis and suppression of tumourigenesis.

In the present study, we provide *in vitro* and *in vivo* evidence that EAF2 mediates apoptosis of GC B cells but not naive B and other immune cell types. EAF2 deficiency causes not only enlarged GC and elevated humoral immune responses but also high susceptibility to collagen-induced arthritis (CIA) and autoantibody production. These findings identify EAF2 as a GC B-specific apoptosis inducer in the immune system that functions to maintain the balance between immunity and tolerance.

## Results

### *Eaf2* is an apoptosis inducer highly expressed by GC B cells

**Figure 1:**
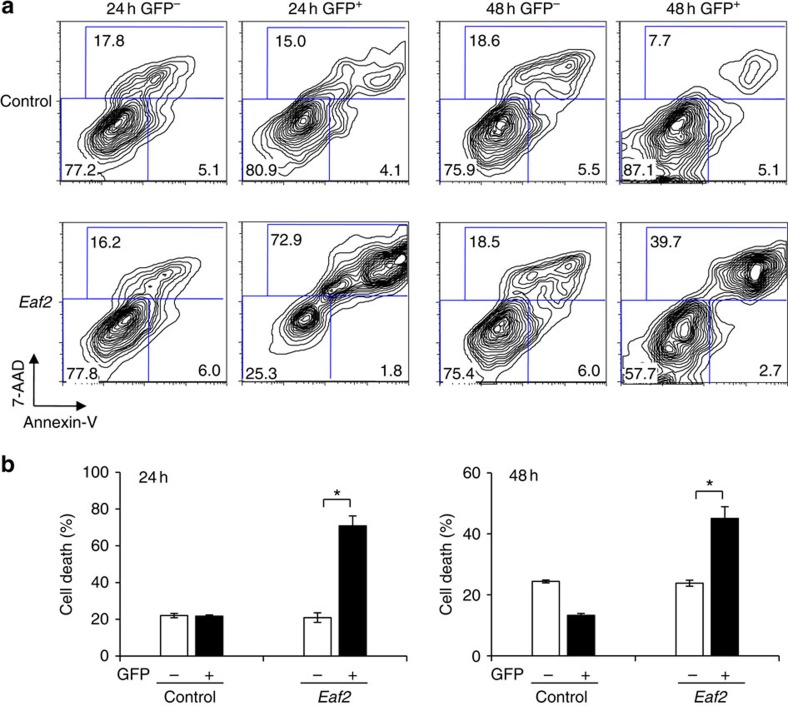
Overexpression of Eaf2 induces the death of normal B cells. Purified spleen B cells (1 × 10^5^ per ml) were stimulated with 10 μg ml^−1^ of LPS for 24 h and then transduced with retrovirus expressing GFP (control) or EAF2-IRES-GFP (Eaf2). The cells were further cultured for 24 and 48 h and analysed for cell death by Annexin-V and 7-AAD staining. (**a**) Representative FACS profiles of B cells cultured for 24 and 48 h. (**b**) Percentages of apoptotic (Annexin-V^+^7-AAD^−^)+dead (7-AAD^+^) cells in gated GFP^−^ and GFP^+^ population. Summary of the results of three independent experiments. *P<0.05 (paired *t*-test).

A comparison of gene expression profiles among various immune cell subpopulation identified *Eaf2*, a gene implicated in the apoptosis of prostate cancer cells^21^, as selectively upregulated in the GC B cells but not in naive B or B cells activated *in vitro* by the various stimuli (Supplementary Fig. 1a), or in spleen T cells before and after T cell receptor stimulation, sorted conventional and plasmacytoid dendritic cells, as well as many other immune cell types (Supplementary Fig. 1b). This expression pattern suggested that EAF2 might be involved in the apoptosis of GC B cells. We therefore first examined whether EAF2 plays a role in B-cell apoptosis. Purified spleen B cells activated with lipopolysaccharide (LPS) were transduced with control green fluorescent protein (GFP) or EAF2-IRES-GFP retrovirus and analysed for cell death in gated GFP^−^ and GFP^+^ cells. As shown in Fig. 1a upper panels, transduction of the control GFP virus did not increase the cell death at either 24 h (left 2 panels) or 48 h (right 2 panels) after virus transduction (compare the virus-transduced GFP^+^ with the non-transduced GFP^−^ population). In contrast, transduction of the EAF2 retrovirus (Fig. 1a lower panels) greatly enhanced cell death at both 24 and 48 h as compared with either virus non-transduced GFP^−^ cells or control virus-transduced cells. These results demonstrate that *Eaf2* overexpression induces B-cell death (Fig. 1b).

### EAF2 specifically mediates the apoptosis of GC B cells

To explore the function of EAF2 *in vivo*, we generated *Eaf2*^−/−^ mice in pure C57BL/6 background (Supplementary Fig. 2a,b). *Eaf2*^−/−^ mice developed normally without any apparent abnormalities and showed no signs of lymphoma and other tumours. Development and maturation of B and T cells appeared normal (Supplementary Fig. 3) and class switch recombination and B-cell proliferation in response to various stimuli were unaffected by EAF2 deficiency (Supplementary Fig. 4a,b). In addition, *Eaf2*^−/−^ mice maintained under specific pathogen-free conditions had normal levels of serum Igs (Supplementary Fig. 4c). Therefore, EAF2 deficiency had no apparent effect on B-cell development and maturation possibly due to its low levels of expression in these cells (Supplementary Fig. 1a).

**Figure 2:**
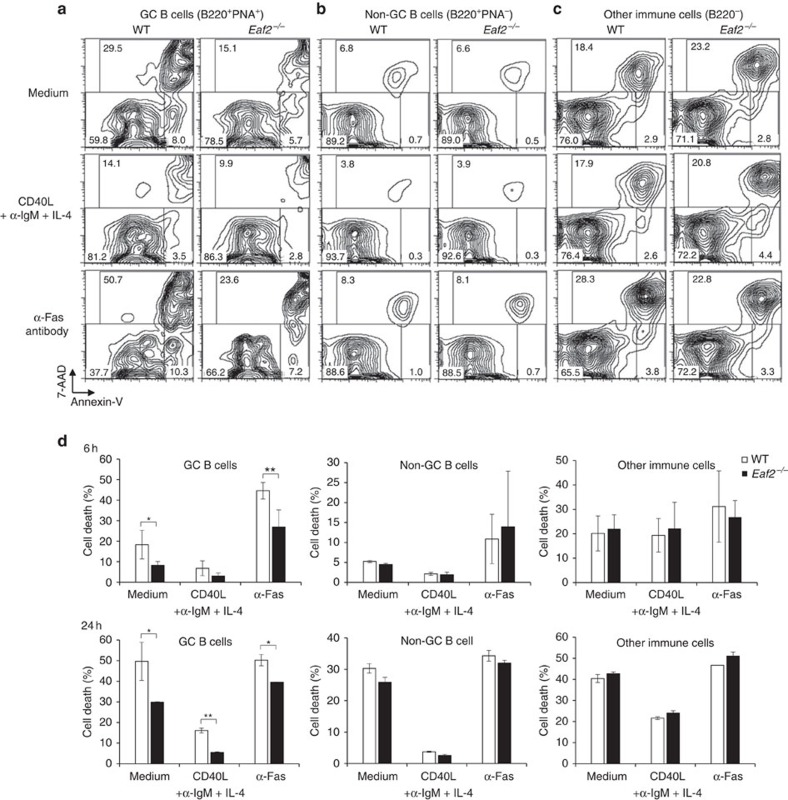
GC B cells from *Eaf2*^−/−^ mice show decreased cell death compared with WT GC B cells. Cells from Peyer's patches (10^6^ cells per ml) were cultured in medium alone or in the presence of CD40L+α-IgM+IL-4 or an agonistic anti-Fas Ab for 6 h and then analysed for cell death as in Fig. 1 in gated GC B cells (**a**), non-GC B (**b**) and other immune cells (**c**). (**d**) Summary of the results obtained with three pairs of WT and *Eaf2*^−/−^ mice. Upper panels, 6 h culture; Lower panels, 24 h culture. White bars, WT; Solid bars, *Eaf2*^−/−^. *P<0.05; **P<0.01 (paired *t*-test).

We next investigated whether GC B-cell death was affected in *Eaf2*^−/−^ mice. Peyer's patches contain a relatively high proportion of GC B cells, as well as mature B and T cells, dendritic cells and macrophages. We therefore used them as a source of GC B and other immune cells, which were cultured under different conditions and analysed for cell death. As shown in Fig. 2a, B220^+^PNA (peanut agglutinin)^+^GC B cells from *Eaf2*^−/−^ mice exhibited significantly reduced cell death compared with those from wild-type (WT) mice either in medium alone (upper panels) or in the presence of the α-FAS Ab (lower panels), which triggers one of the extrinsic pathways of GC B-cell apoptosis^9^,^10^. In the presence of physiological B-cell stimulation conditions (CD40L+α-IgM+IL-4), *Eaf2*^−/−^ GC B cells also showed reduce cell death compared with WT GC B cells at 24 h of culture (Fig. 2d). These observations implicate that EAF2 deficiency not only enhanced spontaneous survival and suppressed FAS-mediated cell death, but also appeared to promote the survival of GC B cells receiving Ag stimulation and T-cell help. In contrast, death of the non-GC B (B220^+^ PNA^−^) cells (Fig. 2b) and other immune cells (B220^−^; Fig. 2c) was not affected by EAF2 deficiency under these culture conditions, an observation consistent with the low levels of EAF2 expression in these cells (Supplementary Fig. 1).

### Enlarged GC with fewer TUNEL^+^ cells in *Eaf2* ^−/−^ mice

**Figure 3:**
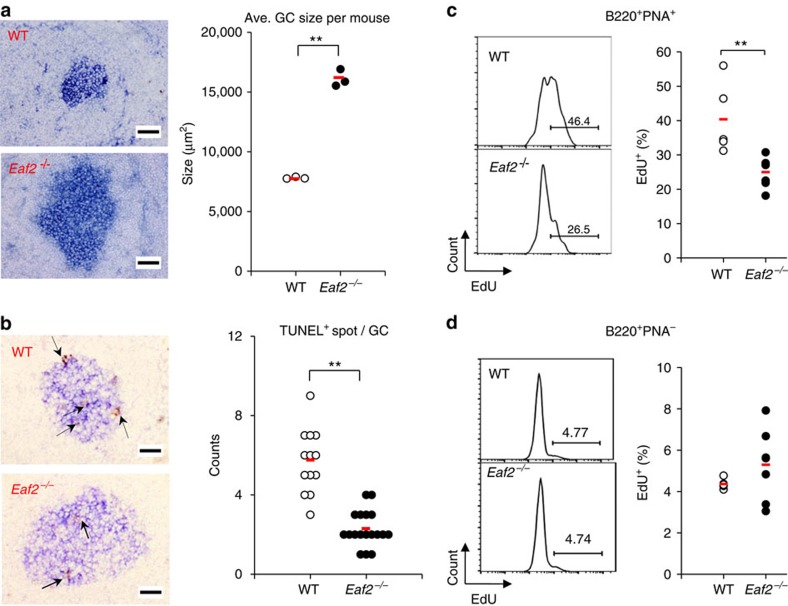
*Eaf2*^−/−^ mice have enlarged GC with decreased apoptosis compared with WT mice. GC formation in the spleen on day 14 after primary immunization with NP–CGG. (**a**) Left, representative PNA staining (blue) of spleen sections in WT and *Eaf2*^−/−^ mice. Right, average size of GC in each mouse. Over 20 GC were measured in each mouse and the average size was calculated. Each symbol represents the average GC size in one mouse. The results of three pairs of WT and *Eaf2*^−/−^ mice are shown. (**b**) Left, representative TUNEL staining (brown spots indicated by arrows are TUNEL^+^), Right, TUNEL^+^ spot numbers per each GC. Brown TUNEL^+^ spots in 7–10 GC of each mouse were counted. The results of two pairs of WT and *Eaf2*^−/−^ mice are shown. For both **a** and **b**, original magnification: × 200. Bars, 50 μm. (**c**) EdU incorporation by B220^+^PNA^+^ GC B cells in WT and *Eaf2*^−/−^ mice. Left, representative FACS profiles of EdU levels; right, results of 5 WT and 6 *Eaf2*^−/−^ mice. (**d**) EdU incorporation by B220^+^PNA^−^ non-GC B cells in WT and *Eaf2*^−/−^ mice. The red bar indicates the mean value of each group. **P<0.01 (unpaired *t*-test).

To investigate whether EAF2 regulates GC B-cell apoptosis under physiological conditions, we next compared GC formation and GC B-cell apoptosis in WT and *Eaf2*^−/−^ mice immunized with the T-dependent (T-D) Ag 4-hydroxy-3-nitrophenyl-acetyl (NP) coupled to chicken γ-globulin (CGG). Two weeks after the immunization, spleen sections were stained with PNA to identify GC B cells^25^. While the average numbers of GC per spleen section were similar between WT and *Eaf2*^−/−^ mice, the GC in *Eaf2*^−/−^ mice were significantly larger than those in WT mice (Fig. 3a, left panels). We measured the sizes of over twenty GC in each mouse and calculated the average size. The results of three pairs of WT and *Eaf2*^−/−^ mice demonstrate that GC in *Eaf2*^−/−^ mice were enlarged by twofold than in WT mice (Fig. 3a, right panel).

To explore whether the enlarged GC in *Eaf2*^−/−^ mice were due to decreased cell death, we next analysed apoptosis in WT and *Eaf2*^−/−^ GC B cells by combining a TUNEL assay and PNA staining (Fig. 3b, left panels). Within GC, the majority of TUNEL^+^ cells/nuclei were contained within tingible body macrophages as clumps of two to five cells^26^. We analysed 7–10 GC per mouse and the results of two pairs of mice revealed a significant reduction of the TUNEL^+^ apoptotic cells in *Eaf2*^−/−^ mice (Fig. 3b, right panel). These results provide physiological evidence that EAF2 is involved in the apoptosis of GC B cells and that lack of EAF2 results in enlarged GC.

The increased survival of GC B cells in *Eaf2*^−/−^ mice may result in an accumulation of non-proliferating B cells. To explore this possibility, we immunized mice with NP–CGG and analysed EdU (5-ethynyl-2′-deoxyuridine) incorporation 2 weeks later. As shown in Fig. 3c, GC B cells (B220^+^PNA^+^) in *Eaf2*^−/−^ mice indeed contained a significantly decreased percentage of EdU^+^ cells than did GC B cells in WT mice. As a control, non-GC B cells (B220^+^PNA^−^) in WT and *Eaf2*^−/−^ mice contained a similarly low percentage of EdU^+^ cells (Fig. 3d). These results suggest that *Eaf2*^−/−^ mice contain an increased proportion of non-proliferating GC B cells.

### Increased numbers of the Ag-specific GC and memory B cells

**Figure 4:**
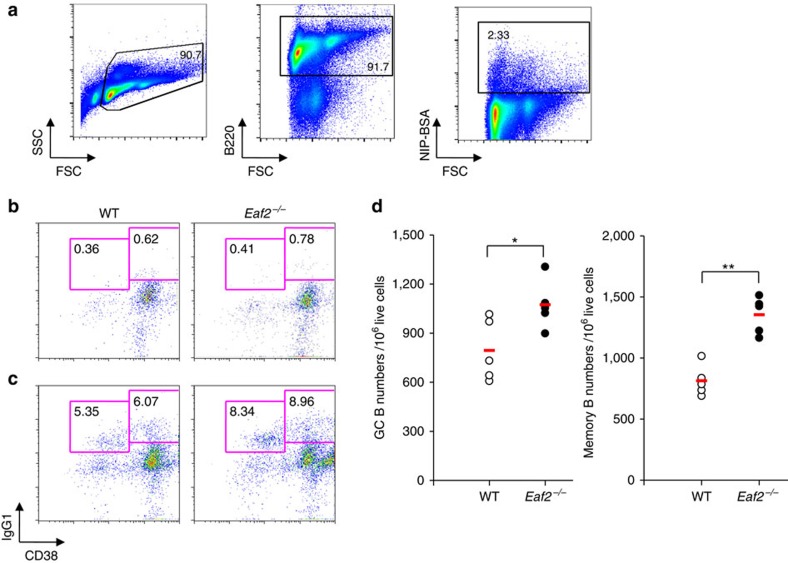
Increased frequency of Ag-specific GC and memory B cells after immunization. Spleen B cells were purified 2 weeks after immunization with NP–CGG and stained with FITC-labelled IgG_1_, PE-conjugated NIP_26_-BSA, APC-conjugated anti-CD38 and PE-Cy7-labelled anti-B220. GC B (NIP binding/B220^+^/IgG_1_^dull^/CD38^dull^) and memory B cells (NIP binding/B220^+^/IgG_1_^high^/CD38^+^) were enumerated. (**a**) Gating strategy for the analysis of GC B and memory B cells. The live B220^+^ NIP-binding cells were analysed for CD38 and IgG_1_ expression. (**b**) Before immunization. (**c**) Two weeks after immunization. Left panel, WT mice; right panel, *Eaf2*^−/−^ mice. (**d**) The tabulated results of five pairs of WT and *Eaf2*^−/−^ mice. Left, GC B cells; right, memory B cells. Open circles, WT mice; Solid circles, *Eaf2*^−/−^ mice. The red bar indicates the mean of five mice. *P<0.05; **P<0.01 (unpaired *t*-test).

We immunized mice with NP–CGG and 2 weeks later stained splenocytes with B220, NIP-BSA, IgG_1_ and CD38 to determine the numbers of NIP-binding (that is, NP-specific) GC B (IgG_1_^dull^CD38^dull^) and memory B (IgG_1_^high^CD38^+^) cells (Fig. 4a–c). At 2 weeks after immunization, both the percentages (Fig. 4c) and the numbers (Fig. 4d) of NP-specific GC B and memory B cells were increased in *Eaf2*^−/−^ mice as compared with WT mice, consistent with the enlarged GC in *Eaf2*^−/−^ mice. However, the numbers of the NP-specific GC B and memory B cells in *Eaf2*^−/−^ mice were reduced to levels similar to those in WT mice at 6 and 9 weeks after immunization (Supplementary Fig. 5a). These observations suggest that the Ag-specific GC B and memory B cells were increased transiently during the peak of the GC reaction but returned to normal levels thereafter.

### Elevated primary immune response in *Eaf2* ^−/−^ mice

**Figure 5:**
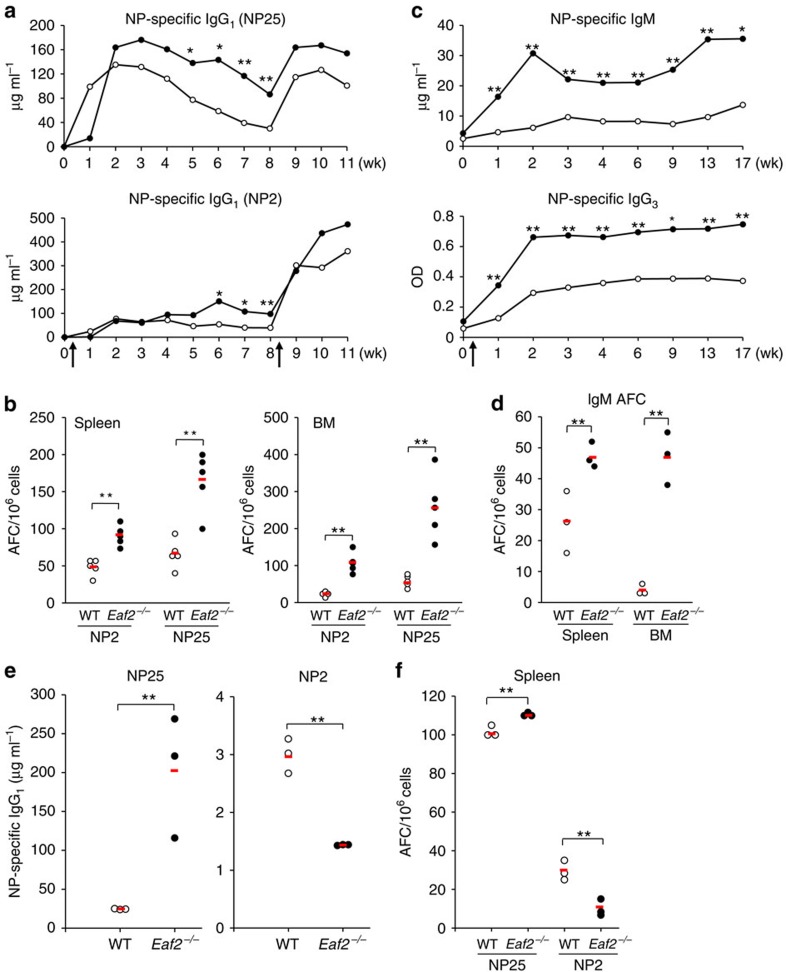
Increased NP-specific Ab in immunized *Eaf2*^−/−^ mice. (**a**) Five pairs of WT and *Eaf2*^−/−^ mice were immunized with 10 μg of NP–CGG in alum. Serum levels of NP-specific IgG_1_ Ab were measured with NP25-BSA (upper) and NP2-BSA (lower) at the indicated time points as described in Methods section. (**b**) AFC in spleen (left) and BM (right) at 6 weeks after NP–CGG immunization. NP2 and NP25 detect high affinity and total NP-specific AFC, respectively. (**c**) NP-specific IgM (upper) and IgG_3_ (lower) serum Ab levels after immunization with NP-Ficoll. The results of five pairs of WT and *Eaf2*^−/−^ mice are shown. (**d**) NP-specific IgM AFC in the spleen and BM at 21 weeks after NP-Ficoll immunization. Three pairs of WT and *Eaf2*^−/−^ mice were analysed. (**e**) and (**f**), *Rag1*^−/−^ mice reconstituted with WT T+WT B or WT T+*Eaf2*^−/−^ B cells were immunized with NP–CGG and analysed for (**e**) NP-specific total IgG_1_ Ab (NP25) and high-affinity IgG_1_ Ab (NP2) in the serum and (**f**) AFC in the spleen. Open circles, *Rag1*^−/−^ mice reconstituted with WT T+WT B cells; Solid circles, *Rag1*^−/−^ mice reconstituted with WT T+*Eaf2*^−/−^ B cells. Arrows in **a** and **c** indicate immunization times. The red bar indicates the mean value of each group. *P<0.05; **P<0.01 (unpaired *t*-test).

The enlarged GC in association with increased Ag-specific GC B and memory B cells in *Eaf2*^−/−^ mice raises the possibility that EAF2 deficiency may cause excessive humoral immune response. Indeed, *Eaf2*^−/−^ mice produced elevated levels of NP-specific total IgG_1_ Ab (measured with NP25) from 2 to 8 wks after immunization with NP–CGG (Fig. 5a, upper panel). The production of the high-affinity NP-Ab (measured with NP2) was also increased in *Eaf2*^−/−^ mice, but from a later time point after immunization (Fig. 5a, lower panel). The elevated production of both total and high-affinity NP-specific Ab was associated with significantly increased numbers of the total and high-affinity Ab-forming cells (AFCs) both in the spleen and bone marrow (BM) (Fig. 5b). When the mice were boosted with NP–CGG in PBS, *Eaf2*^−/−^ mice had an increased Ab response compared with WT mice, but the magnitude of the increase was similar to that observed during the primary response (Fig. 5a). To clarify whether the memory response was affected by EAF2 deficiency, we immunized WT and *Eaf2*^−/−^ mice with NP–CGG in alum and 9 weeks later transferred their spleen B cells together with CGG-primed T cells into *Rag1*^−/−^ mice. Administration of NP–CGG in PBS into the recipient mice revealed similar secondary responses by WT and *Eaf2*^−/−^ memory B cells, as shown by the production of similar amounts of both total and high-affinity NP-specific Ab (Supplementary Fig. 5b) and the generation of similar numbers of the total and high-affinity AFC (Supplementary Fig. 5c). Collectively, the enlarged GC and the increased numbers of the Ag-specific GC B and memory B cells during the peak of GC reaction resulted in increased numbers of AFC and elevated Ab production during the primary response. However, the numbers of both GC B and memory B cells in *Eaf2*^−/−^ mice were reduced to normal levels at later time points during the primary immune response and the memory response was not enhanced by EAF2 deficiency.

We also challenged the mice with the T-independent (T-I) Ag NP-Ficoll. The production of NP-specific IgM and IgG_3_ Ab was significantly increased in *Eaf2*^−/−^ mice as compared with WT mice (Fig. 5c). This increase was sustained for at least 3–4 months after immunization, which was accompanied by increased numbers of NP-specific AFC (Fig. 5d). Although in general T-I Ag do not trigger a strong GC reaction in C57BL/6 mice, it has been reported that NP-Ficoll was able to induce PNA^+^ follicular clusters that resembled GC formed during T-D responses^27^. EAF2 might also mediate apoptosis of such PNA^+^ follicular B cells and/or the proliferating cells outside the follicle that respond to the NP-Ficoll, and its absence could have resulted in increased survival of these cells, leading to elevated T-I Ab responses. Therefore, EAF2 suppresses Ab production to both T-D and T-I Ag for prolonged periods after immunization.

To clarify whether the enhanced Ab production was due to a B-cell-intrinsic defect, purified WT or *Eaf2*^−/−^ spleen B cells were mixed with purified WT spleen T cells and transferred into *Rag1*^−/−^ mice. The recipient mice were then immunized with NP–CGG and analysed for humoral immune responses. *Rag1*^−/−^ mice that received *Eaf2*^−/−^ B cells had significantly elevated production of NP-specific total IgG_1_ Ab (Fig. 5e, left panel) and AFC (Fig. 5f) than those that received WT B cells. Although production of NP-specific high-affinity Ab and AFC was low in the reconstituted mice, *Rag1*^−/−^ mice received *Eaf2*^−/−^ B cells had decreased production of high-affinity Ab (Fig. 5e, right panel) and AFC (Fig. 5f) than those received WT B cells. The frequency of GC B cells appeared to be slightly increased in *Rag1*^−/−^ mice received *Eaf2*^−/−^ B than those received WT B cells (Supplementary Fig. 6a, left panel). The frequency of memory B (Supplementary Fig. 6a, right panel) and T-follicular helper cells (Supplementary Fig. 6b) was similar between *Rag1*^−/−^ mice received either WT or *Eaf2*^−/−^ B cells. These results demonstrate that the enhanced production of NP-specific total Ab and AFC is due to a B-cell-intrinsic defect.

### Ab affinity maturation in *Eaf2* ^−/−^ mice

**Figure 6:**
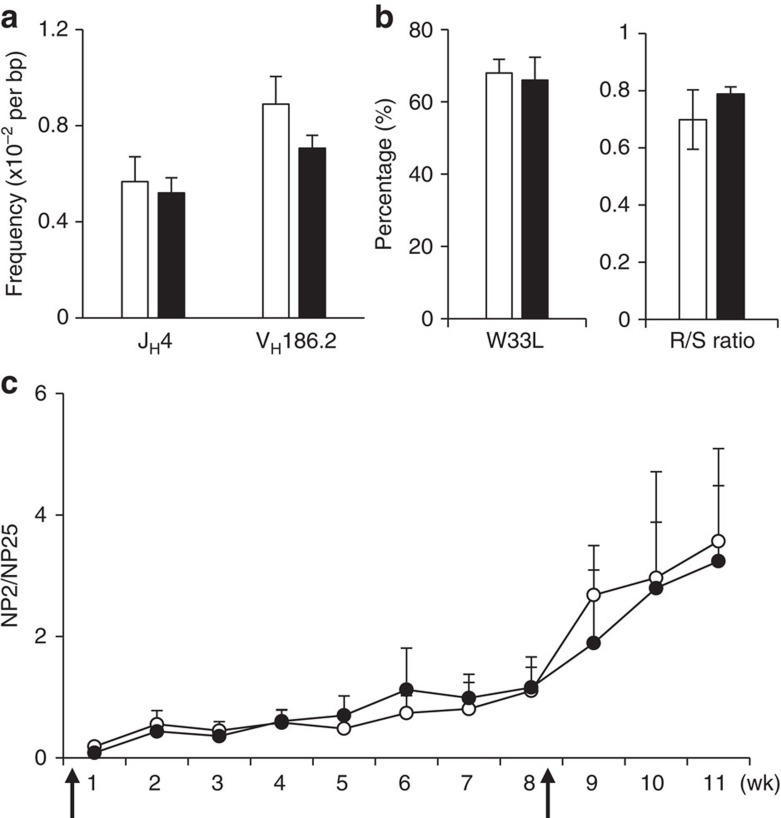
Normal Ab affinity maturation in *Eaf2*^−/−^ mice. (**a**) Mutation frequency in the J_H_4 intronic region and in the *V*_*H*_*186.2* gene in GC B cells isolated from the spleen of WT and *Eaf2*^−/−^ mice immunized with NP–CGG. For the J_H_4 intronic region, 251 and 236 unique clones collected from 3 WT and 2 *Eaf2*^−/−^ mice, respectively, were analysed. For the *V*_*H*_*186.2* gene, 177 WT and 117 *Eaf2*^−/−^ unique sequences form 2 WT and 2 *Eaf2*^−/−^ mice were analysed. (**b**) Left, frequency of the tryptophan to leucine substitution at position 33 (W33L). Right, ratio of aa replacement to silent mutations (R/S ratio) in CDR and FWR. Open bar, WT; Solid bar, *Eaf2*^−/−^ mice. (**c**) Ratio of NP2- and NP25-binding Ab titres in WT (open circles) and *Eaf2*^−/−^ (solid circles) mice, calculated from the data shown in Fig. 5a. The arrows indicate immunization time.

The finding of decreased apoptosis of GC B cells and the enlarged GC during the primary immune response in *Eaf2*^−/−^ mice suggested that Ab affinity maturation might be compromised due to survival of low-affinity B cells. Analysis of SHM in the J_H_4 intronic region, which represents unselected mutations^28^,^29^,^30^, revealed a similar mutation frequency in WT and *Eaf2*^−/−^ mice (Fig. 6a, left two columns). Therefore, EAF2 deficiency did not seem to affect the AID-triggered mutation induction process. We next sequenced the *V*_*H*_*186.2* gene, the primary V gene used in the response to NP in C57BL/6 mice. The frequency and patterns of mutations in the *V*_*H*_*186.2* gene is affected by the selection process for high-affinity Ab in the GC. As shown in Fig. 6a right two columns, the total mutation frequency was slightly decreased in *Eaf2*^−/−^ GC B cells (0.71 versus 0.89% in WT GC B cells). Ab affinity against NP increases ∼10-fold by a single aa substitution (tryptophan to leucine at position 33, W33L) in the complementarity determining region 1. As shown in Fig. 6b left panel, the frequency of W33L was similar between WT (68%) and *Eaf2*^−/−^ (66%) GC B cells, suggesting that GC B cells harbouring this aa substitution were normally positively selected in *Eaf2*^−/−^ mice. In addition, the ratio of aa replacement to silent mutations (R/S ratio) in the complementarity determining region 1+2 region was similar between WT and *Eaf2*^−/−^ GC B cells (Fig. 6b, right panel).

We have also calculated the ratio of NP2- and NP25-binding antibody titres in WT and *Eaf2*^−/−^ mice (Fig. 6c). Although the differences did not reach statistical significance, the ratio of NP2/NP25 was reduced in *Eaf2*^−/−^ mice compared with WT mice during the peak of GC reaction (1–3 weeks of primary responses and after boosting). These observations, along with the significantly decreased production of NP-specific high-affinity Ab and AFC in *Rag1*^−/−^ mice reconstituted with *Eaf2*^−/−^ B cells (Fig. 5e,f) and the moderate reduction of mutation frequency in the *V*_*H*_*186.2* gene (Fig. 6a), collectively suggest a partial impairment in Ab affinity maturation in *Eaf2*^−/−^ mice.

### EAF2 regulates *Bcl-2* and *Bbc3*/*Puma* expression in GC B cells

**Figure 7:**
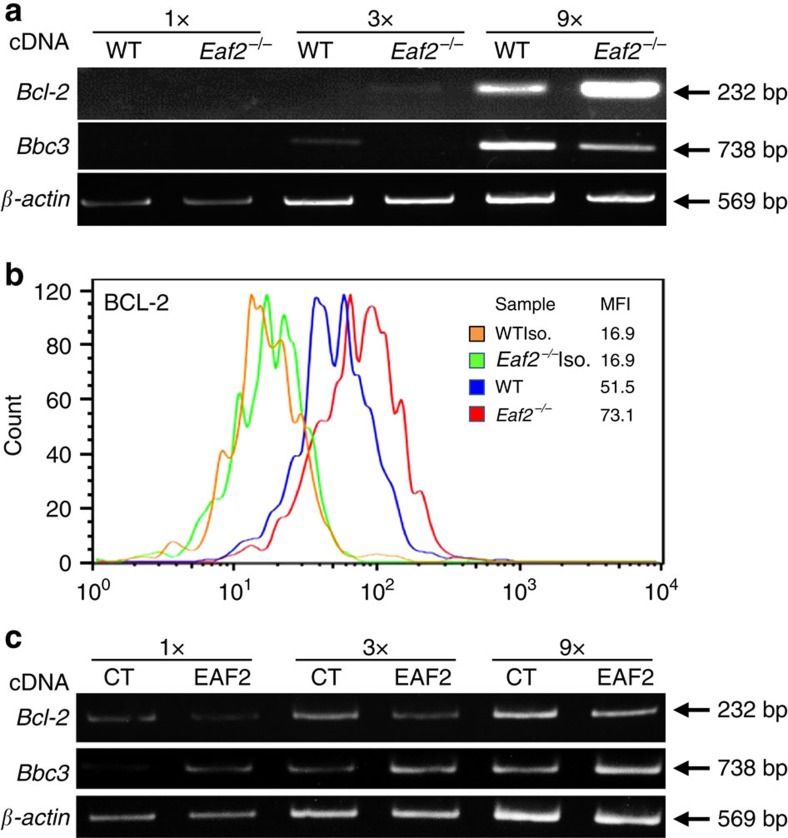
EAF2 regulates *Bcl-2* and *Bbc3*/*Puma* expression in GC B cells. (**a**) Semi-quantitative RT-PCR analysis of *Bcl-2* and *Bbc3* expression in sorted WT and *Eaf2*^−/−^ GC B cells. Increasing amounts of the template cDNA were used. *β-actin* was used as an internal control. (**b**) Intracellular staining of BCL-2 in GC B cells of WT and *Eaf2*^−/−^ mice. Mean fluorescence intensity (MFI) is shown in the upper right. Blue, WT; Red, *Eaf2*^−/−^; Orange and green, isotype control of WT and *Eaf2*^−/−^. Similar results were obtained in three independent experiments. (**c**) Ectopic EAF2 expression downmodulated *Bcl-2* and upregulated *Bbc3* transcription in spleen B cells. Purified spleen B cells were cultured for 24 h in the presence of 10 μg ml^−1^ of LPS and then transduced with retrovirus expressing EAF2-IRES-GFP (EAF2) or GFP alone (CT). Cells were harvested 24 h after retroviral transduction and subjected to RT-PCR analysis. Representative results of 3 experiments are shown.

EAF2 is a transcription elongation-associated factor. To identify potential *Eaf2* target genes in GC B cells, we compared the gene expression profiles between WT and *Eaf2*^−/−^ GC B cells by microarray. Only 268 genes showed significantly differential expression between WT and *Eaf2*^−/−^ GC B cells. A number of apoptosis-related genes, including *Bcl2l10, Bcl6b, Bik, Bag3, Bcl-2, Bbc3, Bcl2a1d, Bcl7c* and *Bcl2l15* genes, were found to be up- or downregulated in *Eaf2*^−/−^ GC B cells. We focused on these apoptosis-related genes and verified their expression by semi-quantitative PCR with reverse transcription (RT-PCR). We found that the transcript level of the anti-apoptotic gene *Bcl-2* was increased while the level of the proapoptotic gene *Bbc3/puma* was decreased in *Eaf2*^−/−^ GC B cells relative to WT GC B cells (Fig. 7a). Furthermore, we confirmed the upregulation of BCL-2 protein expression in *Eaf2*^−/−^ B220^+^PNA^+^ GC B cells by intracellular staining (Fig. 7b). The mean fluorescence intensity of BCL-2 was 68.2±4.4 in *Eaf2*^−/−^ and 56.3±4.2 in WT GC B cells (*P*<0.05, unpaired *t*-test). The transcript levels of the other apoptosis-related genes were either similar between WT and *Eaf2*^−/−^ GC B cells or undetectable in both cells using our semi-quantitative RT-PCR analyses. To further verify that EAF2 regulated *Bcl-2* and *Bbc3/puma* expression, we ectopically expressed EAF2 in spleen B cells. As shown in Fig. 7c, expression of EAF2-IRES-GFP, but not GFP alone (CT), indeed downmodulated *Bcl-2* and upregulated *Bbc3* transcript levels. Moreover, ectopic expression of EAF2 also induced apoptosis in a human Burkitt's lymphoma line Daudi (Supplementary Fig. 7a,b), which again was accompanied by decreased BCL-2 protein expression (Supplementary Fig. 7c) and increased *BBC3* transcript level (Supplementary Fig. 7d). Collectively, these results suggest that EAF2 promotes GC B-cell apoptosis both in mouse and human in part via targeting the expression of *Bcl-2* family genes.

### Loss of EAF2 exacerbated CIA

**Figure 8:**
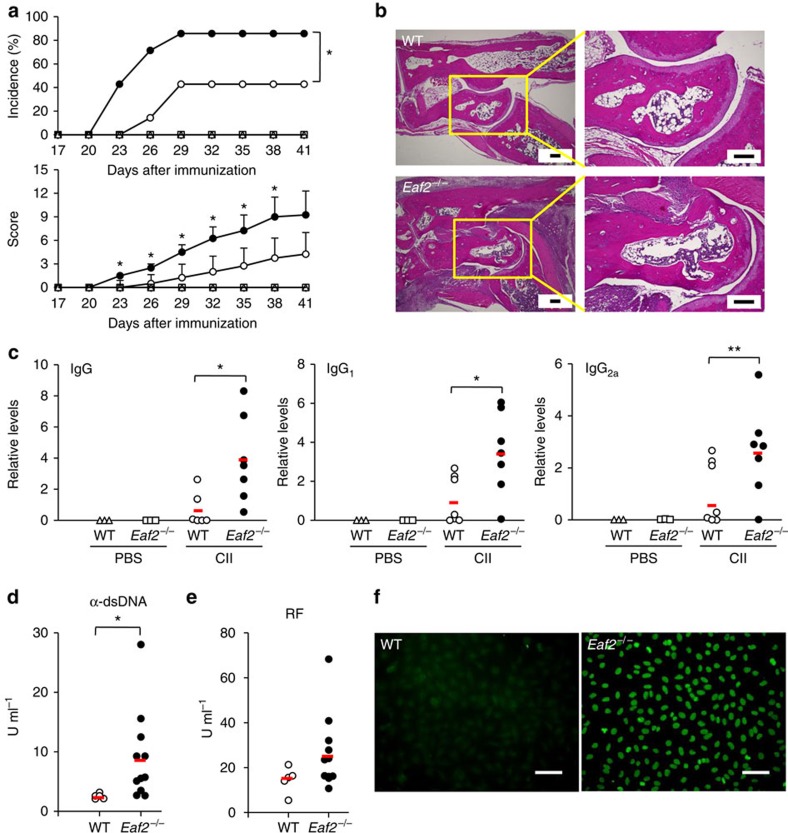
Increased susceptibility to CIA and autoantibody production in *Eaf2*^−/−^ mice. (**a**) Incidence (upper) and clinical scores (lower) of CIA in WT and *Eaf2*^−/−^ mice. Seven pairs of WT and *Eaf2*^−/−^ mice were injected with PBS or immunized with CII on day 0 and day 20. Incidence and clinical scores of CIA were recorded every 3 days between days 20 and 41. Open triangles, PBS-injected WT mice; open squares, PBS-injected *Eaf2*^−/−^ mice; open circles, CII-immunized WT mice; solid circles, CII-immunized *Eaf2*^−/−^ mice. *P<0.05 (upper panel, log-rank test; lower panel, unpaired *t*-test). (**b**) Haematoxylin and eosin (H&E) staining of ankle joints (original magnifications: left, × 40; right, × 100). Bars, 200μm. (**c**) Elevated levels of the CII-specific IgG, IgG_1_ and IgG_2a_ Ab in *Eaf2*^−/−^ mice. The red bar indicates the mean value of each group. *P<0.05, **P<0.01 (unpaired *t*-test) (**d**–**f**) Aged (17 month old) *Eaf2*^−/−^ mice produce increased levels of anti-dsDNA Ab, rheumatoid factor (RF) and ANA. Levels of anti-dsDNA Ab (**d**) and RF (**e**). Open circle, WT; solid circle, *Eaf2*^−/−^ mice. The red bar indicates the mean value of each group. Results of 5 WT and 11 *Eaf2*^−/−^ mice are shown. *P<0.05 (Fisher's exact test). (**f**) ANA. HEp-2 cells (obtained from RIKEN BioResource Center, Japan) were stained with sera from 5 WT and 7 *Eaf2*^−/−^ mice (1:80 dilution). Of five WT mice, only one mouse produced ANA. In contrast, all 7 *Eaf2*^−/−^ mice produced ANA, with six mice showing a homogenous staining pattern and one mouse a centromere pattern (*P*<0.05, Fisher's exact test). Bars, 100 μm. Detailed results are shown in Supplementary Fig. 8a.

SHM can generate self-reactive GC B cells, some of which may not be deleted but differentiate into autoantibody producing plasma cells under certain conditions^31^. The impaired apoptosis of GC B cells in *Eaf2*^−/−^ mice might facilitate the escape of the self-reactive GC B cells from the deletion process, allowing their differentiation into plasma cells secreting self-reactive Abs. This possibility was examined in a CIA model, which is mainly induced by autoantibodies against type II collagen (CII)^32^. We immunized WT (*n*=7) and *Eaf2*^−/−^ (*n*=7) mice with chicken CII in Complete Freund's Adjuvant (CFA) and boosted them on day 20. Three WT and *Eaf2*^−/−^ mice were injected with the same volume of PBS in CFA as a control. By day 3 after the boost, 3 *Eaf2*^−/−^ but none of the WT mice developed arthritis symptoms and by day 9, 6 *Eaf2*^−/−^ but only 3 WT mice showed signs of the disease (Fig. 8a, upper panel). None of the WT or *Eaf2*^−/−^ mice that received PBS in CFA developed arthritis (Fig. 8a). In addition to the increased incidence of arthritis in *Eaf2*^−/−^ mice, their disease severity was also significantly elevated, as shown by the higher clinical scores compared with WT mice (Fig. 8a, lower panel). Histological analysis revealed greater synovial hyperplasia, joint narrowing and bone/cartilage erosion in the joints of arthritic *Eaf2*^−/−^ mice as compared with arthritic WT mice (Fig. 8b).

CIA pathogenesis is largely mediated by CII-specific autoantibodies that bind to cartilage. We therefore determined the levels of anti-mouse CII autoantibodies in the sera on day 41 after the initial immunization. As shown in Fig. 8c, *Eaf2*^−/−^ mice produced 5-, 3.3- and 6.4-fold more CII-specific IgG, IgG_1_ and IgG_2a_ Ab, respectively, than WT mice. No anti-CII Ab was detected in mice injected with PBS in CFA. Collectively, these findings suggest that CII-specific self-reactive GC B cells may not be efficiently eliminated in the absence of EAF2, allowing them to differentiate into plasma cells and produce different classes of CII-specific Ab that contribute to the pathogenesis of arthritis.

### Spontaneous autoantibody production in aged *Eaf2* ^−/−^ mice

We further investigated whether the impaired apoptosis of GC B cells would lead to spontaneous autoantibody production in aged mice even in the absence of deliberate immunization or other external stimuli. As shown in Fig. 8d, WT mice had a mean of 2.5 U ml^−1^ (ranging from 2.1 to 3.2) of anti-dsDNA Ab. In contrast, *Eaf2*^−/−^ mice had a mean of 9.1 U ml^−1^ (ranging from 2.6 to 28.0) of the anti-dsDNA Ab, 8 of 11 *Eaf2*^−/−^ mice had >5 U ml^−1^ and 3 mice had >10 U ml^−1^ of the anti-dsDNA Ab. In addition, *Eaf2*^−/−^ mice also produced an average of 1.8-fold more IgG rheumatoid factor Ab than the WT mice, with 6 of 11 mice producing significantly increased rheumatoid factor levels (Fig. 8e). In fact, 9 of 11 *Eaf2*^−/−^ mice produced elevated levels of either anti-dsDNA or rheumatoid factor, or both. We also examined the anti-nuclear Ab (ANA) in the sera of WT and *Eaf2*^−/−^ mice using the HEp-2 ANA assay. Of the 7 *Eaf2*^−/−^ mice analysed, all produced ANA, with six mice showing a homogeneous staining pattern and one showing a centromere pattern (Supplementary Fig. 8a,f), staining patterns characteristic of autoantibodies from systemic lupus erythematosus and systemic sclerosis patients, respectively. In contrast, only one of five WT mice produced ANA with a homogeneous staining pattern (Supplementary Fig. 8a,f). The titres of total serum IgG were no different between the aged WT and *Eaf2*^−/−^ mice (Supplementary Fig. 8b), indicating that the increased levels of autoantibodies in *Eaf2*^−/−^ mice were not due to generally elevated levels of total serum IgG. These results suggest that *Eaf2*^−/−^ mice accumulate various autoantibodies as they age likely due to the impaired elimination of self-reactive GC B cells.

## Discussion

In the present study, we have identified EAF2, a transcription elongation-associated factor, as an apoptosis inducer for GC B but not naive B and other immune cell types. Absence of EAF2 resulted in enlarged GC accompanied by decreased GC B-cell apoptosis in response to the T-D Ag NP–CGG. In addition, *Eaf2*^−/−^ GC B cells contained an increased proportion of non-proliferating, possibly low affinity and/or Ag-nonspecific, GC B cells. Consistent with these observations, Ab affinity maturation was partially impaired in *Eaf2*^−/−^ mice as shown by a mild reduction in the ratio of high-affinity NP-specific Ab during the peak of GC reaction, a significant decrease in the production of high-affinity Ab in *Rag1*^−/−^ mice received *Eaf2*^−/−^ B cells, and a moderate reduction in the mutation frequency of the *V*_*H*_*186.2* gene. It is intriguing to note while *Eaf2*^−/−^ mice had increased production of both total and high-affinity NP-specific Ab and AFC as compared with WT mice, *Rag1*^−/−^ mice reconstituted with *Eaf2*^−/−^ B cells had increased production of total Ab and AFC but decreased production of high-affinity Ab and AFC compared with those received WT B cells. We think that while *Eaf2*^−/−^ mice contained increased proportion of low-affinity GC B cells, high-affinity GC B cells were able to outcompete low-affinity GC B cells for both Ag binding and presentation to T_FH_ cells, leading to their selective proliferation and differentiation into AFC (refs 33, 34, 35). In contrast, *Rag1*^−/−^ mice reconstituted with purified B and T cells may not allow efficient expansion of the high-affinity GC B cells due to a defective follicular dendritic cell network and/or additional defect in GC formation, resulting in poor generation of high-affinity AFC and Ab. In this scenario the enhanced survival of the low-affinity *Eaf2*^−/−^ GC B cells would result in elevated production of low-affinity AFC and Ab at the expense of the production of high-affinity AFC and Ab.

While the selection for the high-affinity GC B cells was only moderately affected in *Eaf2*^−/−^ mice, the elimination of self-reactive GC B cells appeared to be impaired, as suggested by the finding that *Eaf2*^−/−^ mice produced 3.3–6.4-fold more CII-specific autoantibodies than did WT mice in response to CII immunization. We postulate that GC B cells that have low or no affinity for a foreign Ag need not be actively eliminated by apoptosis since such B cells are unable to compete with the high-affinity GC B cells for the limited amount of foreign Ag to receive a survival and activation signal for their continued expansion. It is the self-reactive GC B cells that need to be actively eliminated by apoptosis to prevent their differentiation into Ab-producing plasma cells.

In addition to the high susceptibility to CIA and the production of increased levels of CII-specific autoantibodies, *Eaf2*^−/−^ mice also produced anti-dsDNA, rheumatoid factor and ANA as they aged even though the mice were maintained under specific pathogen free conditions and in the absence of any additional genetic defect or any extrinsic stimuli such as an infection. Recent studies suggest that ANA arise predominantly from non-self-reactive B cells that are transformed into self-reactive cells by the process of SHM, presumably during the GC reaction^36^,^37^. GC B cells undergo massive apoptosis and some of the apoptotic cells are not efficiently removed. Such apoptotic cells may be the source of self Ag including dsDNA and nuclear Ag (ref. 31). Absence of EAF2 may allow the survival of self-reactive GC B cells that are generated by SHM. Over time, some of these GC B cells might react with self Ag in the GC and differentiate into plasma cells that produce autoantibodies. It remains to be elucidated where and how plasma cells producing self-reactive Ab are generated in *Eaf2*^−/−^ mice. GC reactions are continuously occurring in the intestine in response to bacteria and food Ag and it is conceivable that self-reactive GC B cells can be generated in these structures. Collectively, the present study suggests that EAF2 mediates B-cell apoptosis to eliminate self-reactive GC B cells and this mechanism is critical for the prevention of autoantibody production and autoimmunity.

We have shown that EAF2 is involved in FAS-mediated apoptosis. In agreement with our finding, the phenotypes in *Eaf2*^−/−^ and in *lpr* and B-cell-specific FAS-deficient mice have some similarities^17^,^38^. Both *Eaf2*^−/−^ and FAS-deficient mice have an increased proportion of GC B cells after immunization^17^. In addition, despite the increase of total GC B cells, both mutant mice have relatively normal-affinity maturation in serum Ab. Most importantly, both *Eaf2*^−/−^ and *lpr* mice produce autoantibodies. However, following immunization, *Eaf2*^−/−^ mice have a transient expansion of Ag-specific GC B cells and a prolonged increase in Ag-specific AFC, which are not observed in *lpr* or FAS-deficient mice. Moreover, *lpr* mice have greatly increased numbers of memory B cells during the late primary response with altered GC selection against affinity-enhancing mutants^16^ while *Eaf2*^−/−^ mice only exhibit a moderate and transient increase in the memory B population with comparable GC selection. The differential phenotypes may be also due to the fact that *Eaf2*^−/−^ GC B cells are only partially resistant to FAS-mediated apoptosis. These observations suggest that EAF2 and FAS have overlapping but distinct functions in regulating apoptosis in GC B, AFC and memory B cells.

We found that EAF2 deficiency resulted in increased *Bcl-2* and decreased *Bbc3/puma* expression and conversely ectopic EAF2 expression downmodulated *Bcl-2* and upregulated *Bbc3* expression. Attempts to demonstrate that EAF2 directly targets *Bcl-2* and *Bbc3* genes have been unsuccessful either due to the quality of our EAF2 antibodies or because EAF2 indirectly regulates *Bcl-2* and *Bbc3* transcription. Proteins of the BCL-2 family play a critical role in controlling the humoral immune response^39^. Transgenic mice overexpressing the BCL-2 protein in B cells were characterized by increased numbers of memory B cells and AFC (ref. 11). *Bcl-xL* transgenic mice exhibited fewer apoptotic cells in GC and an increase in the numbers of AFC, especially in spleen, after immunization compared with WT mice^13^. *Eaf2*^−/−^ mice therefore have some similarity to the *Bcl-2*- and *Bcl-xL* transgenic mice in terms of the increased numbers of AFC for prolonged periods. The numbers of Ag-specific GC B and memory B cells are increased only transiently in *Eaf2*^−/−^ mice, which is different from the long-term increase of the Ag-specific memory B cells observed in *Bcl-2*-transgenic mice. Mice deficient in BIM or NOXA showed enlarged GC (refs 35, 40, 41). Loss of BBC3/PUMA resulted in increased memory B cells but did not perturb B-cell activation *in vitro* or GC formation *in vivo*^42^. EAF2 deficiency did not affect the expression of *Bim* and *Noxa* but caused reduced expression of *Bbc3*/*Puma*. Therefore, EAF2 may promote GC B-cells apoptosis and restrict GC sizes through the combined effects of BCL-2 down modulation and *Bbc3*/*Puma* upregulation, as well as regulation of additional apoptosis-related proteins. While Bcl-2 transgenic and BIM-deficient mice developed autoimmunity^43^,^44^, this was accompanied by altered lymphocyte development and strikingly increased numbers of B cells in these mice. In contrast, *Eaf2*^−/−^ mice showed normal lymphocyte development and maturation and only GC B cells exhibited impaired apoptosis. Therefore, *Eaf2*^−/−^ mice represent a unique autoimmune-prone mouse model predominantly caused by the impaired GC B-cell apoptosis.

*In vitro* and *in vivo* functional assays indicated that EAF2 has a tumour suppressive role in prostate cancer^21^. *Eaf2* knockout mice on a mixed genetic background developed lung adenocarcinoma, hepatocellular carcinoma, B-cell lymphoma, and high-grade prostate intraepithelial neoplasia while EAF2 deficiency on a C57BL/6 background developed prostatic intraepithelial neoplasia but lacked macroscopic tumours in other organs^24^,^45^. Consistent with these observations, we have not observed lymphoma or other tumours in our *Eaf2*^−/−^ mice which are generated on a pure C57BL/6 background. Intriguingly, as they aged our *Eaf2*^−/−^ mice had an increased percentage of B220^+^ cells and a higher proportion of large B cells than the WT mice (Supplementary Fig. 9a–c). Southern blot analysis using a J_H_ probe revealed that both WT and *Eaf2*^−/−^ spleen B cells had some retention of the germline band but otherwise showed a smear of bands (Supplementary Fig. 9d), suggesting that there was no obvious monoclonal expansion of pre-leukaemic B-cell clone(s) in these mice. These observations suggest that the impaired apoptosis in *Eaf2*^−/−^ mice resulted in gradual accumulation of polyclonally activated B cells, some of which may undergo malignant transformation depending on the genetic background and/or environmental factors.

In conclusion, we have identified EAF2 as an apoptosis inducer of GC B but not naive B and other immune cell types. Absence of EAF2 resulted in excessive humoral immune responses and autoantibody production, indicating that EAF2 plays an important role in the maintenance of immune balance. Further studies are required to elucidate how EAF2 regulates its target gene expression and induces apoptosis in GC B cells. EAF2 is expressed in human tonsil and Daudi Burkitt's lymphoma cells, as well as in normal B cells and several other immune cell types according to the BioGPS database^46^. In addition, EAF2 was shown to be differentially expressed in lupus patients homozygous for the lupus-associated *MECP2* risk versus protective haplotype^47^ and it will be of interest to explore the role of EAF2 in the development and/or progression of human autoimmune diseases.

## Methods

### Retrovirus transduction

Naive spleen B cells were purified from C57BL/6 mice by using a B cell negative purification kit (IMag beads, 557792, BD Biosciences). Purified B cells were cultured in the presence of 10 μg ml^−1^ LPS for 24 h and then transduced with retrovirus expressing GFP alone or EAF2-IRES-GFP as described previously^48^. Briefly, 4 μg of pMX-IRES-GFP or pMX-EAF2-IRES-GFP constructs were transfected into 1.5 × 10^6^ packaging cells using Lipofectamine (11668-019, Thermo Fisher Scientific) and 48 h later the culture supernatant containing the retrovirus was collected by centrifuging at 8,000 g for 16 h. The precipitated retrovirus was resuspended in 1 ml of culture medium (without antibiotics) and 0.4 ml of the retrovirus was added to 1 × 10^5^ cells in 24-well plates and centrifuged for 1 h at 32 °C, 2,000 r.p.m. The cells were further cultured for 24 and 48 h, followed by staining with Annexin-V and 7-amino-actinomycin D (7-AAD; 559763, BD Biosciences) to detect apoptotic and dead cells, respectively.

### Construction of the Eaf2 targeting vector

The targeting vector was constructed by using a MunI-MunI (3047-bp) and a BamHIII-ApaI (5090-bp) genomic fragment as 5′ and 3′ homologous regions, respectively, to replace *Eaf2* exon 1 containing the translation initiation ATG codon with a neomycin (*neo*) gene flanked by *loxP* sites.

### Generation of *Eaf2* ^−/−^ mice

The *Eaf2* targeting construct was linearized with PvuI and electroporated (250 V, 500 μF) into C57BL/6-derived Bruce4 embryonic stem cells. Two days after the transfection, the embryonic stem cells were cultured in the presence of 600 μg ml^−1^ of G418 (G7034, Sigma) and 2 μM of ganciclovir (078–04481, WAKO Chemicals, Japan; only for the first 2 days) for positive and negative selection of the targeted cells, respectively. Correctly targeted embryonic stem cell clones were microinjected into C57BL/6 blastocysts, and the embryos were transferred into foster mothers. Chimeric mice were generated and bred with C57BL/6 mice to obtain heterozygotes, which were further bred to obtain homozygotes. The mice were maintained under specific pathogen-free conditions in the animal facilities of the RIKEN Center for Integrative Medical Sciences and the Institute of Medical Science, the University of Tokyo. Protocols approved by the Animal Studies Committees of RIKEN Yokohama Institute and the University of Tokyo were used for all animal experiments.

### RT-PCR analysis

For *Eaf2* expression, primers s214 and as562 were used and the PCR was performed at 95 °C for 2 min followed by 30 cycles of 95 °C for 10 s, 55 °C for 20 s and 72 °C for 1 min. For mouse *Bcl-2* and *Bbc3/puma* expression, the following primer sets were used: *Bcl-2*/s809, *Bcl-2*/as1040, *Bbc3/puma*/s759 and *Bbc3/puma*/as1496. PCR was performed at 95 °C for 2 min followed by 35 cycles of 95 °C for 10 s, 60 °C for 10 s and 72 °C for 1 min. For human *BBC3/PUMA* expression, h*BBC3*/s791 and h*BBC3*/as1339 primers were used. PCR was performed at 95 °C for 2 min followed by 35 cycles of 95 °C for 10 s, 60 °C for 10 s and 72 °C for 1 min. The primers sequences are listed in Supplementary Table I.

### *In vitro* culture of Peyer's patch cells

GC cells isolated from Peyer's patches of three pairs of WT and *Eaf2*^−/−^ mice (16 weeks old) were added to 96-well plates (1 × 10^6^ cell ml^−1^, 100 μl per well) and cultured in medium alone, or in the presence of CD40L (threefold dilution of culture supernatants from a myeloma cell line producing soluble CD40L (ref. 49)), 5 μg ml^−1^ F(ab')_2_-α-IgM (16-5092-85, eBioscience) plus 20 ng ml^−1^ IL-4 (404-ML-010, R&D systems), or 1 μg ml^−1^ agonistic α-FAS Ab (554254, BD Biosciences). Six and 24 h later, cells were collected and stained with FITC-PNA (FL-1071, Vector Laboratories, 1600x dilution), APC-B220 (103211, Biolegend, 200x dilution), and Annexin-V and 7-AAD (559763, BD Biosciences) to detect apoptotic and dead cells.

### Spleen histology

Three pairs of WT and *Eaf2*^−/−^ mice (12 weeks old) were killed on day 14 after immunization with NP–CGG (N-5055C-5, Biosearch Technologies), and their spleens were removed and embedded in Tissue Tek OCT compound (23-730-571, Fischer Scientific) by flash-freezing in liquid N_2_. Blocks of frozen tissue were stored at −80 °C until sectioning^50^. Spleens were cut on a cryostat as 5-μm-thick sections and thaw mounted onto MAS-coated slides. Sections were allowed to air dry for 10 min, and stored at room temperature until use. We analysed spleen sections from the largest circumference, and measured the sizes of >20 GCs from each mouse to determine the average GC size.

### PNA staining of spleen sections

Before immunohistochemical staining, spleen sections were fixed in ice-cold acetone for 10 min. Endogenous peroxidase activity was blocked by 10 min room temperature incubation in 0.3% H_2_O_2_ before staining. Sections were stained in 300-fold diluted biotinylated PNA (B-1075, Vector Laboratories) for 1 h in a humidified chamber, followed by Streptavidin-alkaline phosphatase (AP) (7100-04, SouthernBiotech). Bound AP was then visualized by enzymatic detection with 0.125 mg ml^−1^ of naphthol-AS-MX phosphate (855-20 ML, Sigma), 0.25 mg ml^−1^ Fast Blue BB salt (44670, Sigma) and 2 mM levamisole (1359302, Sigma) in 0.1 M Tris-HCl (pH 8.5)^8^. Ten min later, stained sections were washed in PBS and mounted in 30% glycerol.

### TUNEL assay

Two pairs of WT and *Eaf2*^−/−^ mice (12 weeks old) were immunized and their spleen sections were prepared as described for spleen histology and PNA staining. Endogenous peroxidase activity was blocked by 10 min room temperature incubation in 0.3% H_2_O_2_ before staining, followed by washing with PBS. Sections were then incubated for 60 min in a 37 °C humidified incubator with terminal deoxynucleotidyl transferase enzyme in the presence of reaction buffer (S7100, Apoptag *In situ* detection kit, Chemicon International)^51^. After washing three times with PBS, digoxigenin was added on the section for 30 min in a 37 °C humidified incubator. Sections were counterstained with PNA as described above. Horseradish peroxidase (HRP)-labelled TUNEL^+^ cells were visualized using 0.4 mg ml^−1^ 3-amino-ethyl-carbazole (A6926, Sigma) and 0.005% H_2_O_2_ in 0.05 M sodium acetate buffer (pH 5.0). TUNEL^+^ cells were counted at a magnification of × 200 by systematic scanning of the entire section. Counts were confirmed by blinded recounts.

### EdU incorporation assay

EdU incorporation was performed using a Click-iT EdU Alexa Fluor kit (C10419, ThermoFisher) according to the manufacturer's instruction. Briefly, 5 WT and 6 *Eaf2*^−/−^ mice (10 weeks old) were immunized with NP–CGG and 12 days and 13 days later injected intraperitoneal (i.p.) with 200 μl of (1 mg ml^−1^) EdU. EdU incorporation was analysed on day 14 by FACS.

### Detection of NP-specific GC and memory B cells

B cells were purified from spleen of age-matched immunized WT and *Eaf2*^−/−^ mice (five pairs, 10–11 weeks old) by negative sorting using a B-cell enrichment set (557792, BD Biosciences). Purified spleen B cells were stained with FITC-labelled IgG_1_ (406607, Biolegend, 100x dilution), PE-conjugated NIP_26_-BSA (a kind gift from Dr Takemori, 500x dilution), APC-conjugated anti-CD38 (303510, Biolegend, 40x dilution) and PE-Cy7-labelled anti-B220 (561881, BD Biosciences, 80x dilution). GC B cells (NIP binding/B220^+^/IgG_1_^dull^/CD38^dull^) and memory B cells (NIP binding/B220^+^/IgG_1_^high^/CD38^+^) were analysed by FACSCalibur (BD Biosciences).

### Immune response

For T-D Ab production, five pairs of WT and *Eaf2*^−/−^ mice (8 weeks old) were injected i.p. with 10 μg of NP–CGG in alum and boosted with 10 μg of NP–CGG in PBS 8-weeks later. For T-I Ab production, five pairs of WT and *Eaf2*^−/−^ mice (8 weeks old) were immunized with 10 μg of NP-Ficoll (F-1420, Biosearch Technologies). Mice were bled weekly from the retro-orbital sinus and the serum titres of NP-specific IgG_1_, IgM or IgG_3_ were analysed. For measuring NP-specific high and total (high and low affinity) Ab, plates (NUNC AIS, Denmark) were coated with 50 μl per well of 2.5 μg ml^−1^ of NP2-BSA (N-5050L, Biosearch Technologies, NP2 plate) and 2.5 μg ml^−1^ of NP25-BSA (N-5050H, Biosearch Technologies, NP25 plate), respectively, in PBS overnight, followed by blocking with 1% BSA in PBS. After washing with 0.05% Tween 20 in PBS, sera at various dilutions were added and the plates were then incubated for 1 h at room temperature. The bound immunoglobulin was detected using HRP-conjugated goat Ab specific to each mouse Ig isotype (SouthernBiotech) and developed for 20 min using an ABTS solution. Serial dilutions of the control high (clone C6) and low (clone N1G9) affinity NP-specific Ab were included to determine the concentrations of NP2-binding (high affinity) and NP25-binding (total) Ab, respectively. Since the high-affinity Ab are known to bind to NP25-BSA inefficiently, the amount of NP-specific total Ab (measured with NP25-BSA using N1G9 as a control) was likely underestimated.

For determination of anti-collagen II Ab, immunosorbent plates were coated with 5 μg ml^−1^ of chicken type II collagen (20012, Chondrex) in 10 mM acetic acid overnight. After blocking with 10% BSA in 0.05% T-PBS for 2 h, sera serially diluted with 0.5% BSA in 0.05% T-PBS were added into the plate and incubated for 2 h at room temperature. After washing, HRP-conjugated goat anti-mouse IgG, IgM, IgG_1_ or IgG_2a_ Ab were added and incubated for 1 h at room temperature, and 1-Step Ultra TMB ELISA substrate (34028, Thermo) was added to each well. The colour reaction was stopped with 1.2 M H_2_SO_4_ and the absorbance at 450 nm wavelength was measured.

### ELISPOT assay

ELISPOT assay was performed using a Multiscreen HTS filter plate (Millipore). The HTS plate was coated with 50 μg ml^−1^ of NP2-BSA or NP25-BSA at 4 °C overnight. The coated plate was then washed three times with PBS-T (PBS containing 0.1% Tween 20) and blocked with PBS containing 1% BSA for 1 h at room temperature. Splenocytes (3 × 10^5^, 1.5 × 10^5^ and 0.75 × 10^5^) were then seeded and incubated at 37 °C for 100 min in a CO_2_ incubator. The plate was then washed twice with PBS-T containing 50 mM EDTA, three times with PBS-T and blocked again with PBS containing 1% BSA for 1 h at room temperature. The plate was further incubated with AP-conjugated goat anti-mouse IgG_1_ Ab (1 μg ml^−1^ in PBS containing 1% BSA) at 37 °C for 60 min in a CO_2_ incubator, washed four times with PBS-T and developed with BCIP/NBT reagent (MOSS INC) for 2–3 min. The plate was then washed four times with H_2_O, air dried and colonies were counted using an IMMUNOSPOT Analyzer (CTL Analyzers LLC). The number of colonies obtained with different numbers of splenocytes was converted to number of colonies per 10^6^ cells and the mean values are shown.

### Adoptive transfer

These experiments were performed as described previously^48^. Briefly, spleen B cells were purified from 5 WT or 5 *Eaf2*^−/−^ mice (10 weeks old) and spleen T cells were purified from 3 WT mice (10 weeks old). 1 × 10^7^ B cells were mixed with 5 × 10^6^ T cells and transferred into 9-week-old *Rag1*^−/−^ mice of C57BL/6 background (CLEA Japan). One week after the transfer, the recipient mice were immunized i.p. with 100 μg of NP–CGG and analysed for humoral immune responses 2 weeks later.

### Sorting of the GC B cells

WT and *Eaf2*^−/−^ mice (9 weeks old) were immunized with 100 μg of NP–CGG. Two weeks later, the splenic cells were stained with PE-B220 (103208, Biolegend, 200x dilution) and FITC-PNA and the B220^+^PNA^+^ GC B cells were sorted using a FACSAria (BD Biosciences). Genomic DNA was isolated for the analysis of Ig gene SHM and total RNA was isolated for gene expression analysis.

### Induction of CIA

CIA was induced as previously described^32^. Briefly, 9-week-old sex-matched WT (C57BL6/J) and *Eaf2*^−/−^ mice (generated on a pure C57BL6/J background) were injected intravenously with 100 μl of a 1/1 (v/v) emulsion of 0.05 M acetic acid containing 100 μg of chick type II collagen (20012, Chondrex) and CFA (7023, Chondrex). The day of the first immunization was defined as day 0. Twenty days after the primary immunization, the mice were boosted with 100 μg of collagen II in IFA via the same route. The severity of arthritis was evaluated every 3 days after the boost by visual inspection. Each paw was scored for clinical signs of arthritis as described^52^. The clinical score for each mouse was the sum of the four paw scores, which results in a maximum score of 16. On the final day of the experiments, all mice were anaesthetized and their blood was collected by cardiac puncture.

### Histopathologic analysis

After completing the CIA experiments, mice were killed and hind paws were fixed in 4% phosphate-buffered paraformaldehyde solution, decalcified in Kalkitox (112–00651, Wako, Japan), and then embedded in paraffin. The tissues were longitudinally cut into 3-μm serial sections and stained with haematoxylin and eosin. The histopathological changes in the joints were examined under light microscopy.

### Detection of autoantibodies

For measuring the levels of anti-dsDNA and rheumatoid factor, anti-dsDNA (AKRDD-061, Lbis) and rheumatoid factor IgG (AKRRG-101, Lbis) mouse ELISA kits were used. For measuring ANA, mouse sera diluted at 1:80 were tested for ANA using a HEp-2 kit (ORG870, Orgentec). The HEp-2 slides were incubated with the diluted sera for 30 min followed by washing with PBS. Subsequently, the slides were stained with 1 μg ml^−1^ of Alexa Fluor-488-goat anti-mouse IgG (H+L) Ab (A11029, Life technologies) and visualized using a fluorescence microscope.

### Statistical analysis

Statistical significance was assessed by an unpaired or paired *t-*test, Fisher's exact test, or log-rank test (*P<0.05; **P<0.01).

## Additional information

**How to cite this article:** Li, Y. *et al*. EAF2 mediates germinal centre B-cell apoptosis to suppress excessive immune responses and prevent autoimmunity. *Nat. Commun.* 7:10836 doi: 10.1038/ncomms10836 (2016).

## Supplementary Material

Supplementary Information Supplementary Figures 1-9 and Supplementary Table 1
