# Supplementary material for: EAF2 mediates germinal centre B-cell apoptosis to suppress excessive immune responses and prevent autoimmunity
Source: Nat Commun. 2016 Mar 3;7:10836. doi: 10.1038/ncomms10836 (PMC4782062; doi:10.1038/ncomms10836)
Supplement: Supplementary Information — Supplementary Figures 1-9 and Supplementary Table 1 [file ncomms10836-s1.pdf]

Supplementary information

EAF2 mediates germinal center B cell apoptosis to suppress excessive immune responses and prevent autoimmunity.

Yingqian Li, Yoshimasa Takahashi, Shin-ichiro Fujii, Yang Zhou, Rongjian Hong, Akari Suzuki, Takeshi Tsubata, Koji Hase and Ji-Yang Wang

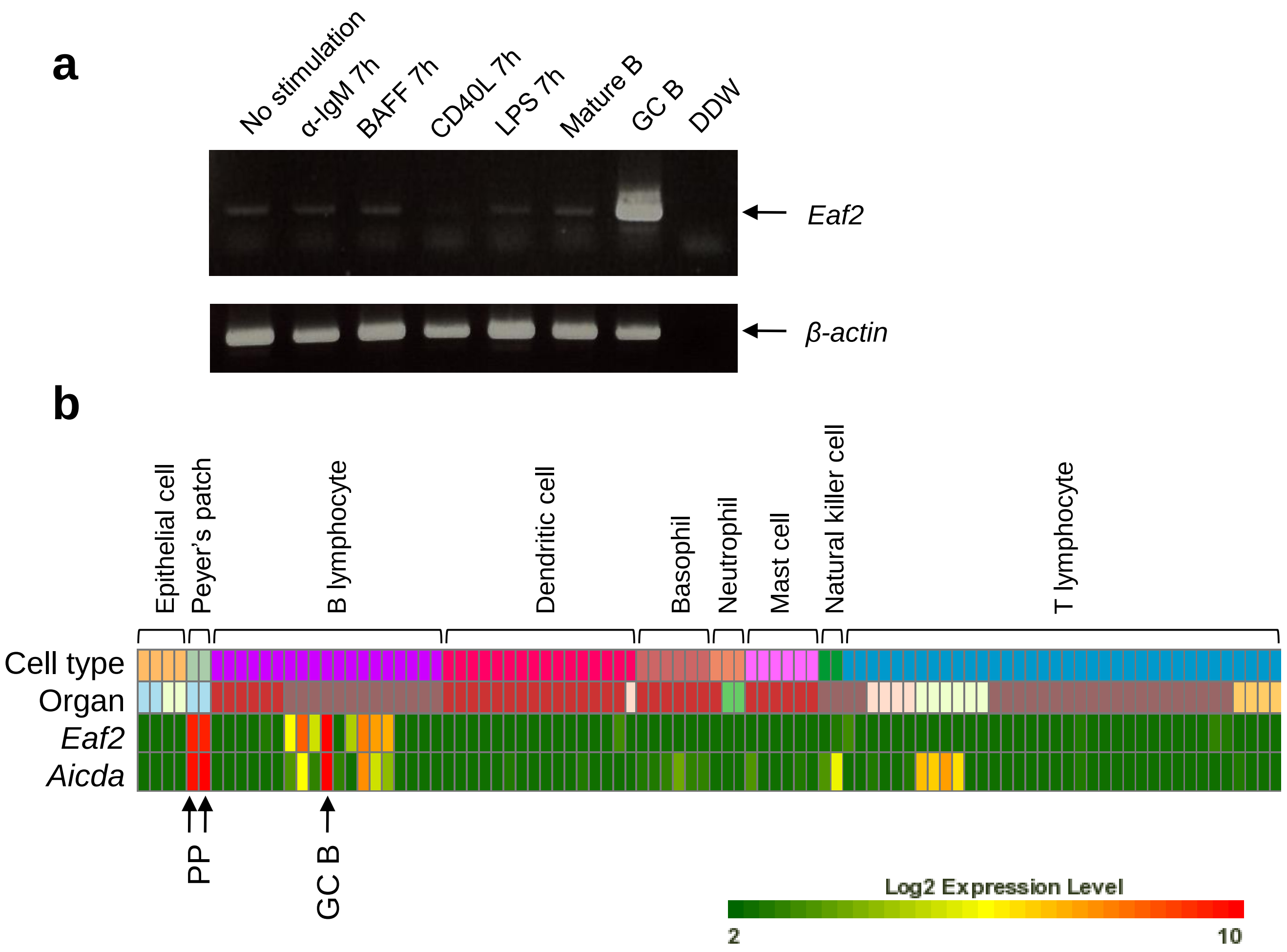

Supplementary Figure 1. (**a**) ***Eaf2* is specifically upregulated in GC B cells**. Purified spleen B cells were cultured in medium alone (No stimulation) or in the presence of indicated stimuli. GC B cells were sorted from mice 2 wks after immunization with NP-CGG in alum. Purified spleen B cells without culture (Mature B) were also included in the analysis. Total RNA was extracted using Trizol reagent (Invitrogen) and first-strand cDNA was synthesized with *Superscript III* reverse transcriptase and random primers. RT-PCR was performed as described in Materials and Methods. (**b**) **Microarray analysis of *Eaf2* expression in 94 different immune cell types/subpopulation.** The results of *Aicda* (Activation-induced cytidine deaminase), which is specifically expressed by GC B cells, are shown as a reference. The expression pattern of *Eaf2* is quite similar to that of *Aicda*, being highly expressed in Peyer's patches (PP) and sorted GC B cells (arrows).

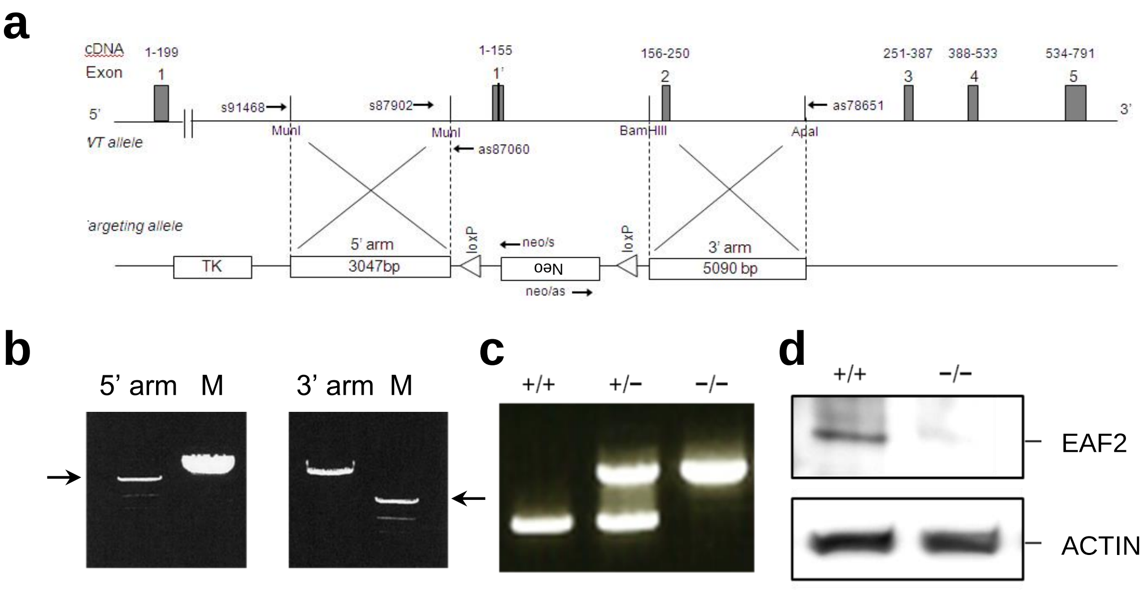

Supplementary Figure 2. Generation of *Eaf2*$^{-/-}$ mice. (**a**) Targeting strategy. The targeting vector was designed to replace exon 1' and the upstream promoter region with a neo gene. The positions of PCR primers are indicated. (**b**) Long-range PCR analysis to detect the correctly targeted allele, using primers S91468 and neo/s for the 5'-arm and as78651 and neo/as for the 3'-arm. The amplification was performed under the following conditions: 95 ° C for 2 min followed by 30 cycles of 95 ° C for 10 s, 55 ° C for 20 s and 72 ° C for 7 min. The predicted sizes for the 5'- and 3'-PCR were 4.07-and 6.05-kb (indicated by an arrow), respectively. M, marker. (**c**) Mouse genotypes were screened by PCR using primers s87902, as87060 and neo/s. The PCR was performed at the following conditions: 95 ° C for 2 min followed by 30 cycles of 95 ° C for 5 s, 55 ° C for 10 s and 72 ° C for 2 min 30 s. (**d**) Immunoblot analysis of EAF2 protein expression in spleen B cells purified from WT and *Eaf2*$^{-/-}$ mice.

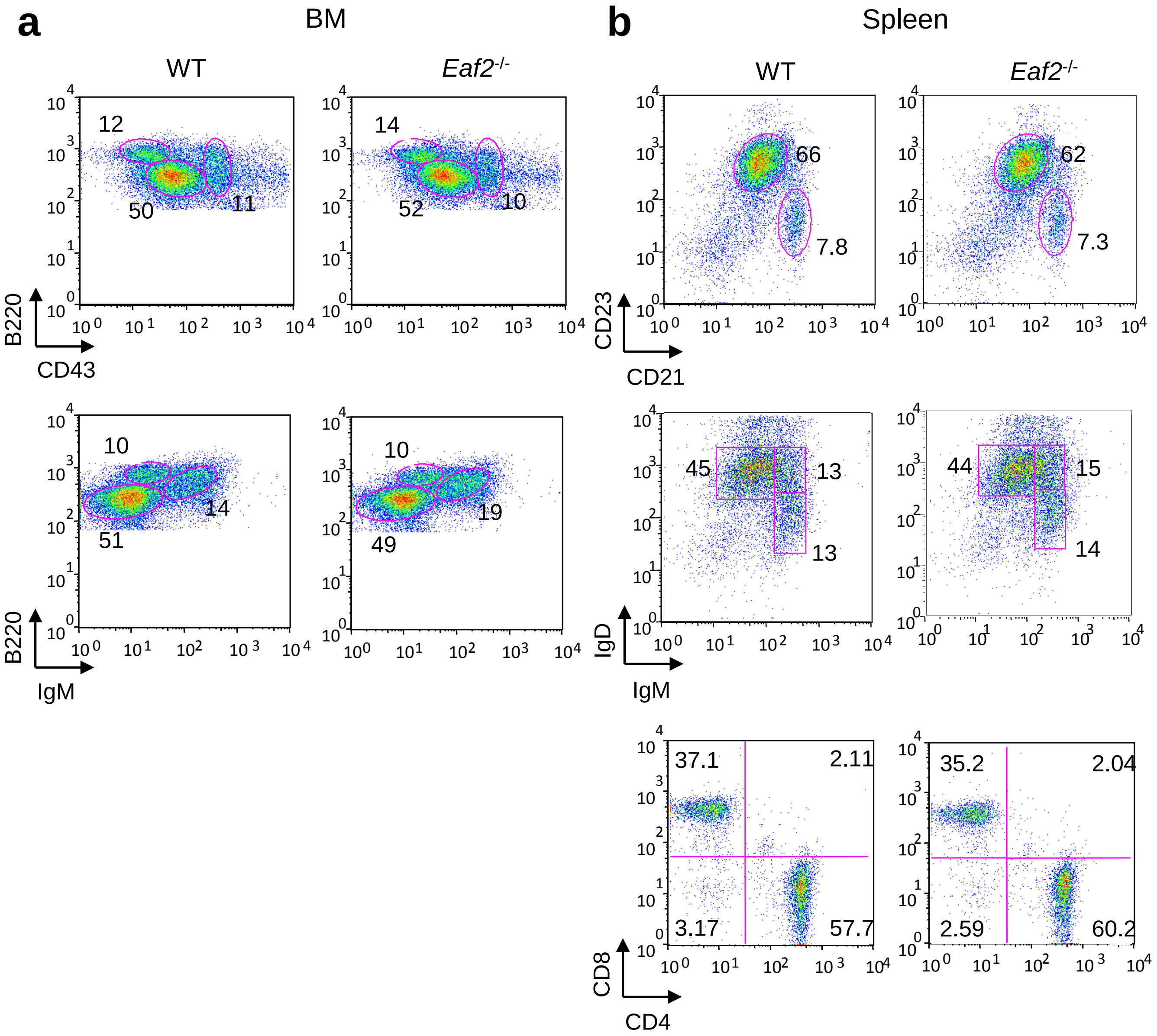

Supplementary Figure 3. Development and maturation of B and T cells in WT and *Eaf2*$^{-/-}$ mice. (**a**) BM cells were analyzed by flow cytometry for B220 vs. CD43 or IgM expression. (**b**) Profiles of CD23 vs. CD21 (upper) and IgD vs. IgM (middle) in gated B220^{+} spleen cells. Lower panels: CD8 and CD4 expression in gated TCR^{+} spleen cells.

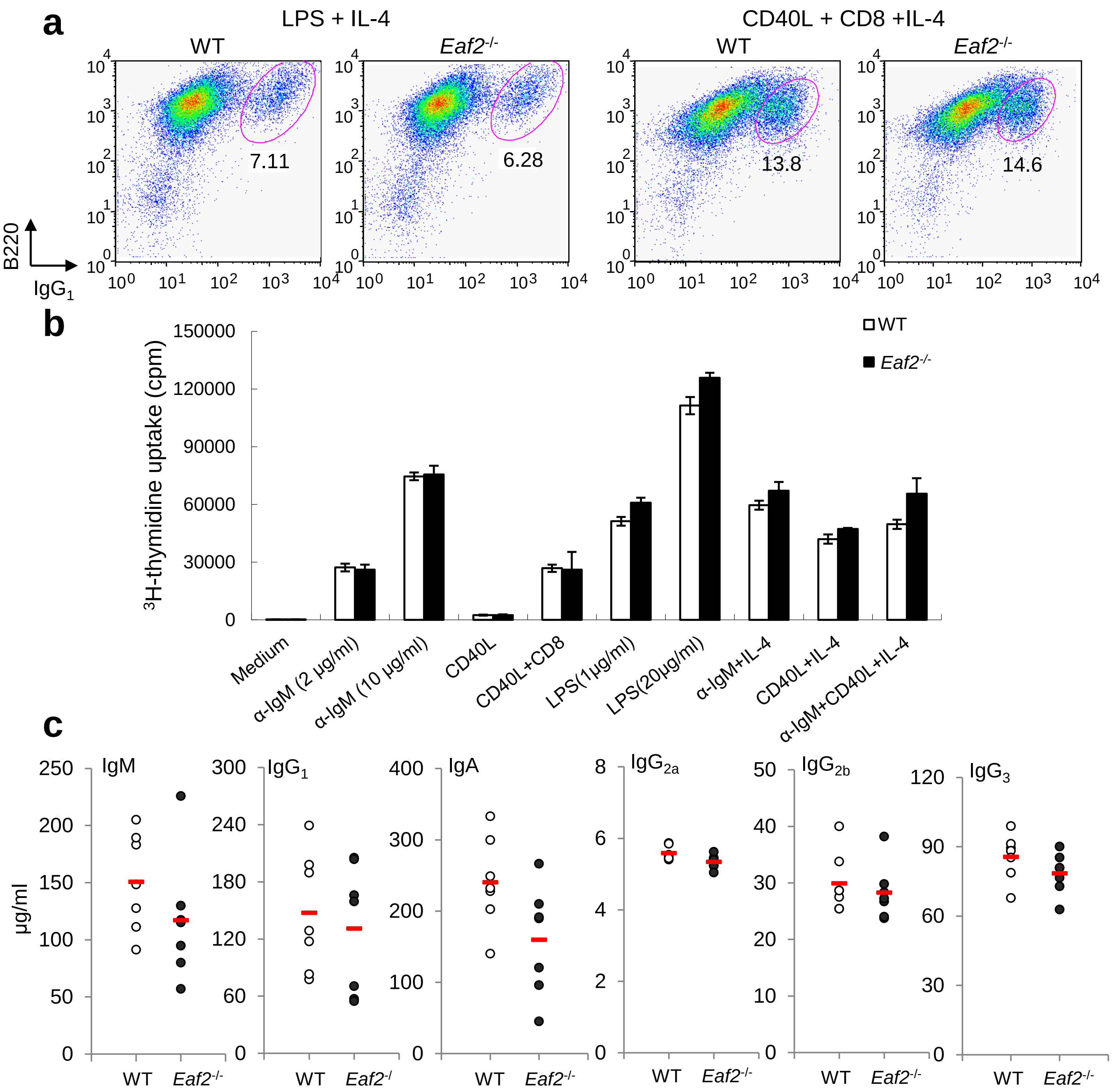

Supplementary Figure 4. Normal class switch recombination (CSR), *in vitro* responses and serum Ig levels in *Eaf2*$^{-/-}$ mice. (**a**) Purified spleen B cells (5x10^5/ml, 1 ml/well in a 12-well plate) were cultured for 48 h in the presence of LPS+IL-4 or CD40L+IL-4 and analyzed for B220 and IgG_1 expression. (**b**) Proliferative responses. Purified spleen cells (5x10^5/ml, 100 μl/well in 96 flat-bottom plates) were cultured for 48 h in medium alone or in the presence of different doses of α-IgM antibodies, 1/3 dilution of CD40L-CD8α fusion protein (CD40L), CD40L crosslinked with an α-CD8α antibody (CD40L+CD8), LPS, or various combination of these stimuli and pulsed with ^{3}H-thymidine for the last 6 h. (**c**) Seven pairs of age-matched WT and *Eaf2*$^{-/-}$ mice (9–10 weeks old) were bled, and serum Ig levels were measured by ELISA. Open circles, WT; solid circles, *Eaf2*$^{-/-}$ mice. The red bar indicates the mean value of 7 mice.

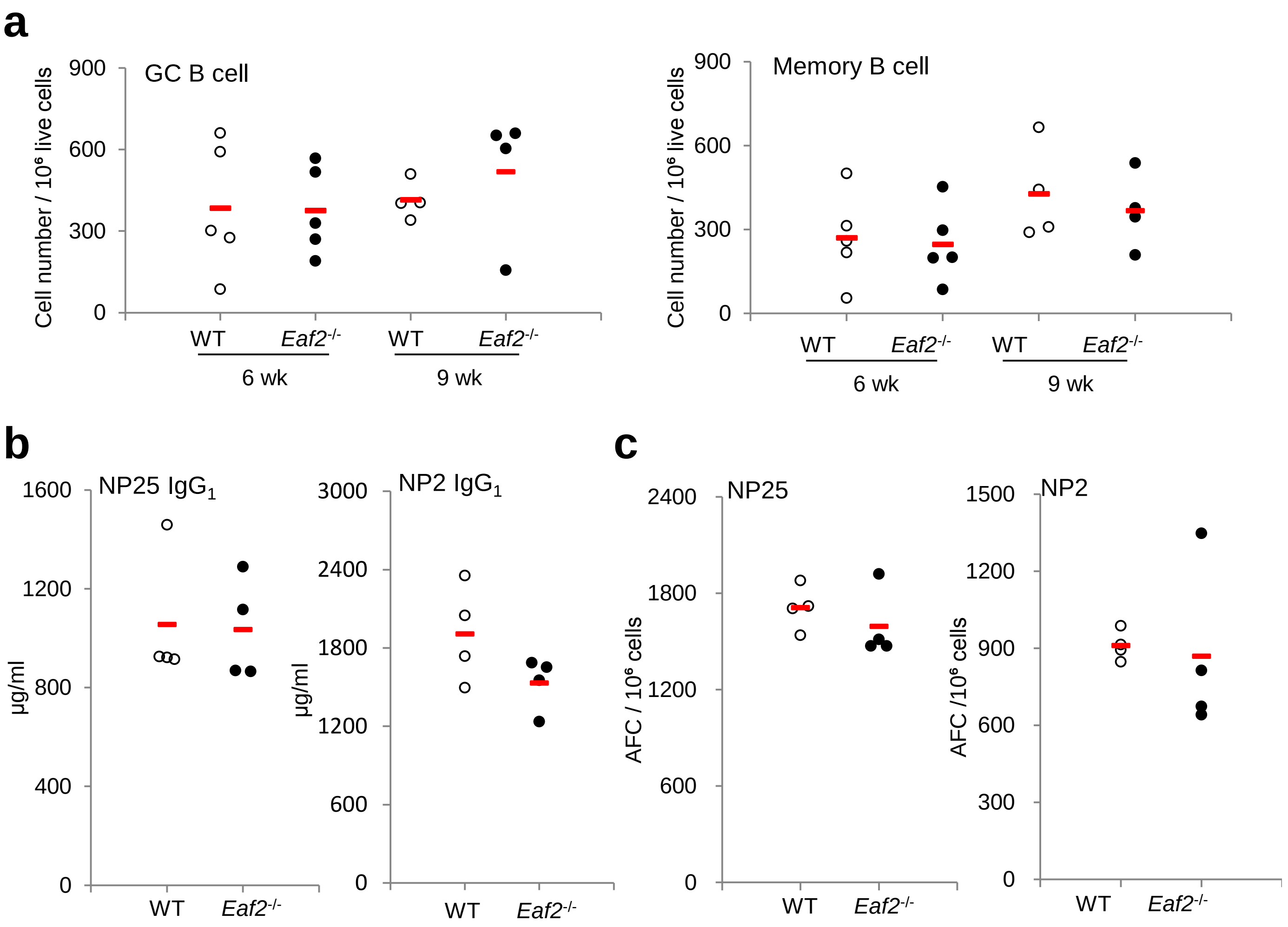

Supplementary Figure 5. Normal memory responses in *Eaf2$^{-/-}$* mice. (**a**) GC B (left) and memory B (right) cells were enumerated 6 and 9 weeks after immunization. (**b**) and (**c**) Normal secondary responses by *Eaf2$^{-/-}$* memory B cells. WT and *Eaf2$^{-/-}$* mice were immunized with NP-CGG and 9 wks later spleen B cells were purified. The purified spleen B cells (2×10^7) were mixed with 1×10^7 of CGG-primed T cells and transferred *i.v.* into *Rag1$^{-/-}$* mice. The recipient mice were injected *i.p.* with 20 μg of NP-CGG in PBS next day and 10 days later analyzed for the total (measured by NP25) and high-affinity (measured by NP2) NP-specific Abs in the sera (**b**) and the total and high-affinity NP-specific AFC in the spleen (**c**). Open circles, WT; solid circles, *Eaf2$^{-/-}$* mice. A red bar indicates the mean value of 4-5 mice.

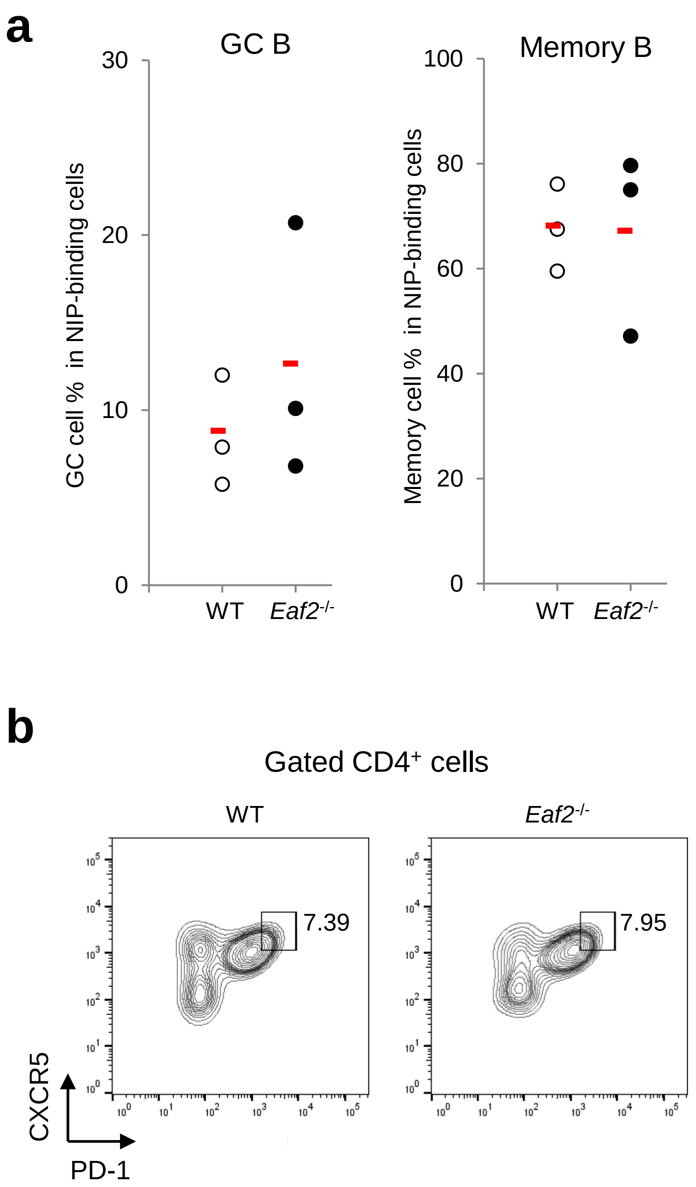

Supplementary Figure 6. Frequency of GC B, memory B and T-follicular helper cells in reconstituted *Rag1*$^{-/-}$ mice. WT B or EAF2-deficient B cells were mixed with WT T cells and transferred into *Rag1*$^{-/-}$ mice. The recipient mice were then immunized with NP-CGG and analyzed 2 weeks later. (**a**) Frequency of GC B (left) and memory B (right) cells. Open circles, WT B cells; Solid circles, *Eaf2*$^{-/-}$ B cells. (**b**) Frequency of T-follicular helper cells.

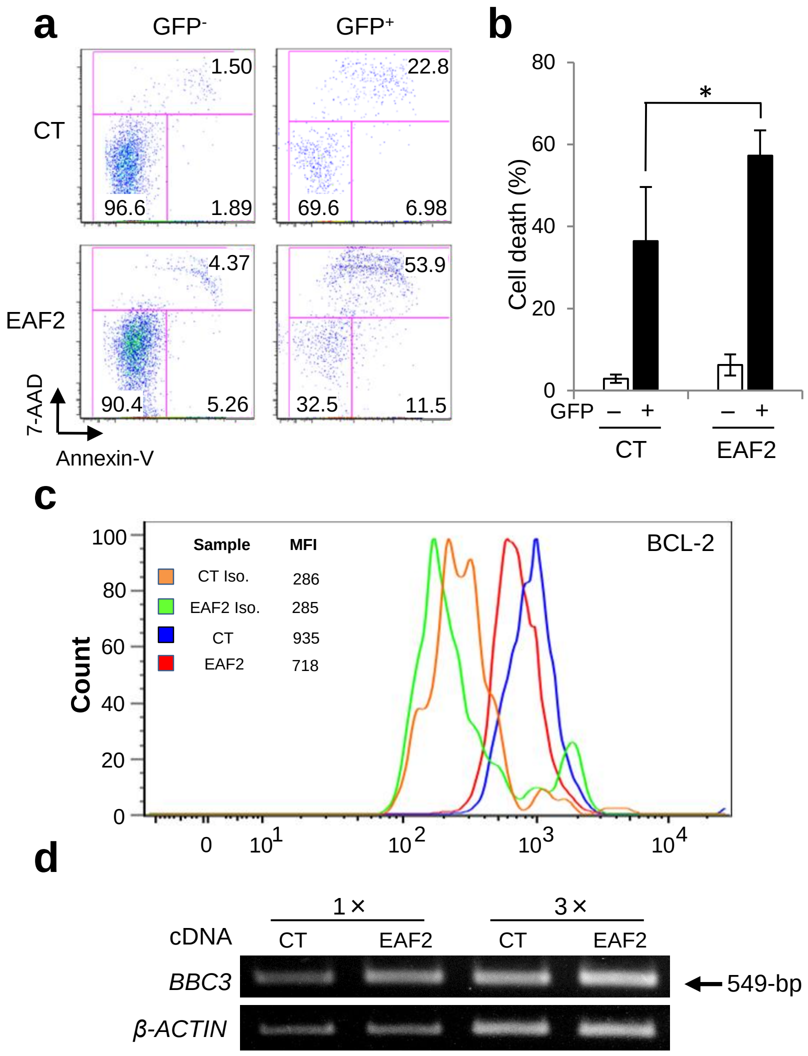

Supplementary Figure 7. EAF2 induced apoptosis and modulated BCL-2 and *BBC3* expression in human Burkitt's lymphoma line Daudi. Daudi cells (obtained from RIKEN BioResource Center, Japan) were transduced with retrovirus expressing EAF2-IRES-GFP (EAF2) or GFP alone (CT) and 24 h later analyzed for apoptosis (**a** and **b**), BCL-2 protein levels by intracellular staining (**c**) and *BBC3* transcript levels (**d**). *$P < 0.05$ (unpaired t-test).

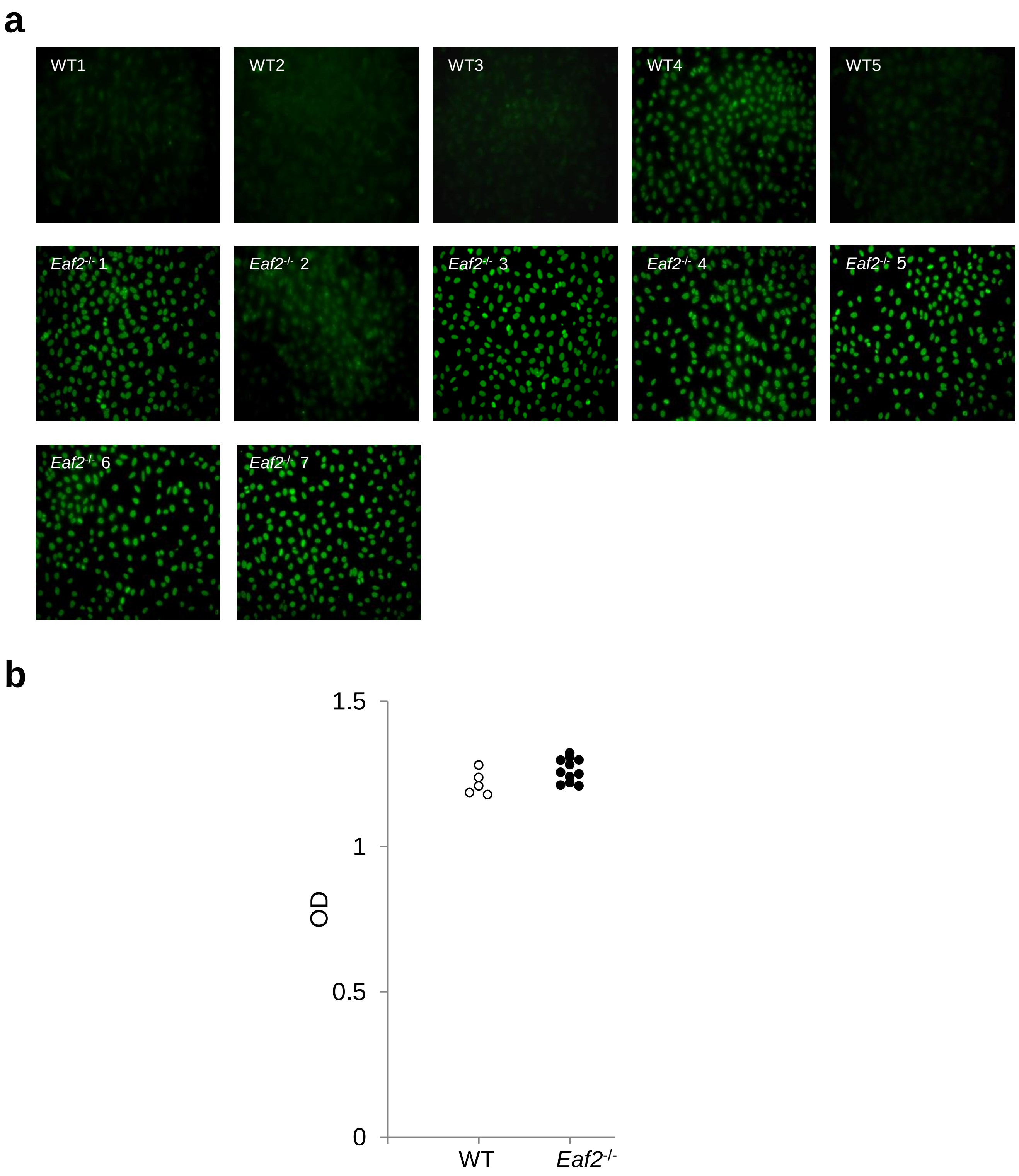

Supplementary Figure 8. *Eaf2$^{-/-}$* mice produce anti-nuclear Abs (ANA). (**a**) Five WT and seven *Eaf2$^{-/-}$* mice were analyzed for ANA production as described in Methods. (**b**) *Eaf2$^{-/-}$* mice have normal levels of total serum IgG. Five WT and eleven *Eaf2$^{-/-}$* mice (17-month old) were bled, and serum IgG levels were measured by ELISA. Open circles, WT; solid circles, *Eaf2$^{-/-}$* mice.

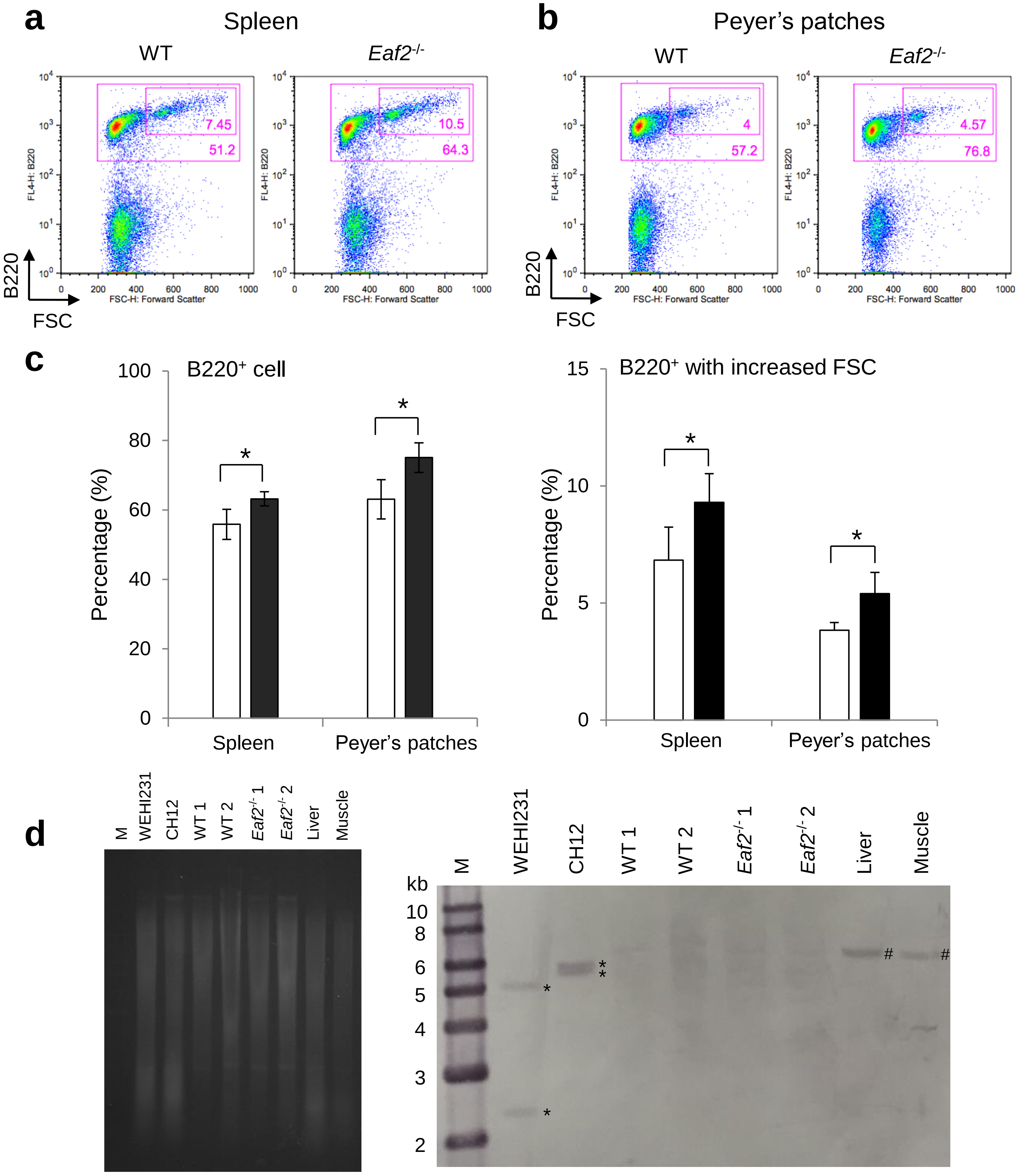

Supplementary Figure 9. Increased B220$^+$ cells in aged *Eaf2*$^{-/-}$ mice. Three pairs of 69-wk-old WT and *Eaf2*$^{-/-}$ mice were sacrificed and their spleens and lymph nodes were analyzed for B220 expression. Representative FACS profiles of spleen (**a**) and Peyer's patches (**b**). (**c**) Left panel, average percentage of B220$^+$ cells; Right panel, average percentage of B220$^+$ cells with increased cell sizes (FSC). White bars, WT mice; Solid bars, *Eaf2*$^{-/-}$ mice. *$P < 0.05$ (unpaired t-test). (**d**) Analysis of B cell clonality. Genomic DNA isolated from WEHI231 and CH12 B lymphoma cells, purified spleen B cells of 2 pairs of WT and *Eaf2*$^{-/-}$ mice, and liver and muscle, was digested with *Eco*RI and resolved in a 0.7% agarose gel (left panel). The resolved DNA was transferred to a nylon membrane and hybridized with a mouse J$_H$ probe (right panel). M, 1-kb ladder; *, rearranged bands; #, 6.4-kb germline band.

Supplementary Table 1. Primers used in genomic and RT-PCR analysis

s91468	5’-GTGTGACTGGAGAAATGGCT-3
as78651	5’-ACCCCAATCCAACCTAACT-3’
neo/s	5’-TCGCCTTCTATCGCCTTCTT-3’
neo/as	5’-ATAGCCGAATAGCCTCTCCA-3’
s87902	5’-GCACGCTTTCTCCTACTTCA-3’
as87060	5'-GTCTTGATTGGCTGTCTGGA-3'
s214	5’-AGGTGAACAGGTGACAATAA-3’
as562	5’-TATCACAACTACTCATCTGG-3’
Bcl-2/s809	5’-GATGGTGTGGTTGCCTTA-3’
Bcl-2/as1040	5’-GGTATATCCGCTACAAGTTAC-3’
Bbc3/puma/s759	5’-GAGACAAGAAGAGCAGCAT-3’
Bbc3/puma/as1496	5’-GAGCACAGGATTCACAGT-3’
h*BBC3/*s791	5’-TCATGGGACTCCTGCCCTTA-3’
h*BBC3/*as1339	5’-AGGCTAGTGGTCACGTTTGG-3’
